# Programmable CRISPR-Cas9 microneedle patch for long-term capture and real-time monitoring of universal cell-free DNA

**DOI:** 10.1038/s41467-022-31740-3

**Published:** 2022-07-09

**Authors:** Bin Yang, Jilie Kong, Xueen Fang

**Affiliations:** Department of Chemistry and Institutes of Biomedical Sciences, Fudan University, Shanghai, 200433 PR China

**Keywords:** Biomedical engineering, Sensors and biosensors, CRISPR-Cas systems, Biosensors, Bionanoelectronics

## Abstract

Recent advances in biointerfaces have led to the development of wearable devices that can provide insights into personal health. As wearable modules, microneedles can extract analytes of interest from interstitial fluid in a minimally invasive fashion. However, some microneedles are limited by their ability to perform highly effective extraction and real-time monitoring for macromolecule biomarkers simultaneously. Here we show the synergetic effect of CRISPR-activated graphene biointerfaces, and report an on-line wearable microneedle patch for extraction and in vivo long-term monitoring of universal cell-free DNA. In this study, this wearable system enables real-time monitoring of Epstein-Barr virus, sepsis, and kidney transplantation cell-free DNA, with anti-interference ability of 60% fetal bovine serum, and has satisfactory stable sensitivity for 10 days in vivo. The experimental results of immunodeficient mouse models shows the feasibility and practicability of this proposed method. This wearable patch holds great promise for long-term in vivo monitoring of cell-free DNA and could potentially be used for early disease screening and prognosis.

## Abstract (web summary)

Real-time sensing of biomarkers via the use of wearable devices is a major aim of personalised medicine. Here, authors demonstrate an on-line wearable microneedle patch for real-time capture and monitoring of universal cell-free DNA.

## Introduction

With the arrival of the third medical revolution, wearable technology has experienced great developments in the field of modern personalized medicine. It is expected that wearable technology will become more streamlined, provide flexibility, and be integrated into personal life^1^. Wearable technology is a multidisciplinary subject that mainly includes soft materials, flexible batteries, wireless signal communication, and chem-biosensing, which have garnered great attention for their ability to provide in-depth medical diagnostics and facilitate personal health assessments^2^. Although most efforts have focused on the analysis of small molecules or electrolytes^3–7^, the next generation of wearables aims for non-invasive or minimally invasive detection of macromolecular biomarkers (e.g., proteins or DNA)^2,4^. However, the greatest challenge for such wearables may be the effective extraction and real-time in vivo monitoring of these biomarkers.

Interstitial fluid (ISF) is an ideal candidate for wearable chem-biosensors^4^. ISF contains a variety of biomarkers (e.g., protein, cells, nucleic acids) that are closely related to blood concentration^2^. As an important component of wearable devices, microneedles (MNs) have been applied to ISF extraction in a safe, painless, and efficient manner^8–10^. In our previous studies^11,12^, we proposed hydrogel MN patches to extract DNA from ISF and then analyzed them with portable flexible electrochemical microfluidics. However, this approach was just the half of story, and still an offline monitoring method and would not be competent to the application of integrated wearable devices in real-world use. Although offline wearable sensors with high sensitivity appear to be widely used for biomarker detection, frequent operation and turnaround times probably inhibit their use for real-time monitoring of disease biomarkers, which is very important in the clinical applications. The real-time wearables for disease-derived biomarker detection not only could offer in-depth information of health and wellness over time for disease progression, but also improve self-management for those intensive care patients countered with abnormal or unpredictable situations. Therefore, the development of an online wearable system that can perform both sample extraction and real-time monitoring would be very important and could significantly improve personal health management.

Clustered regularly interspaced short palindromic repeats (CRISPR) technology has attracted extensive attention for the rapid analysis of nucleic acids with high specificity and accuracy due to its precise gene editing ability under the guidance of programmable single guide RNA (sgRNA) or CRISPR RNA (CrRNA)^13,14^. Unlike zinc-finger nuclease and transcription activator-like effector nuclease technology, CRISPR uses CRISPR-associated (Cas) effectors to recognize and edit specific gene sites, providing promising methods for sequence-specific detection, such as HUDSON-SHERLOCK^15,16^ and DETECTR^17^. Recently, various amplification-free analytical strategies based on CRISPR have been developed for nucleic acids^18–21^. Hajian et al. proposed a graphene field-effect transistor based on CRISPR-Cas9 for unamplified samples, with a detection limit of 1.7 fM^20^. Subsequently, Fozouni et al. constructed an enhanced amplification-free CRISPR-Cas13a platform with multiple CrRNAs for SARS-CoV-2 detection, with a higher sensitivity of 100 copies/μL^21^. Amplification-free CRISPR methodology could provide an effective and simple method for online nucleic acid wearable devices.

In this study, we propose CRISPR-Cas9 activated graphene biointerfaces on conductive MNs and combine them with reverse iontophoresis for the extraction and real-time monitoring of nucleic acids. Owing to the synergetic effect of CRISPR-Cas9 and graphene biointerfaces, the CRISPR-based wearable system is employed for real-time monitoring of Epstein-Barr virus^22,23^, sepsis, and kidney transplantation cell-free DNA (cfDNA). This system has potential application value for long-term real-time monitoring and early diagnosis of cfDNA-derived diseases.

## Results

### Components, principle, and properties of the online CRISPR wearable patch

Here, we demonstrated an online CRISPR-Cas9 activated wearable patch based on the synergetic effect of CRISPR technology and graphene biointerfaces, where conductive MNs and reverse iontophoresis were employed for efficient extraction and real-time monitoring of different cfDNA in a minimally invasive fashion. A promising development in the study is the specific, continuous, and direct monitoring of unamplified target DNA without preamplification (e.g., PCR or HCR). The CRISPR-activated wearable system includes the following modules: a flexible substrate, namely, a modified PDMS membrane; target cfDNA enrichment control, namely, a printed carbon nanotube (CNT)-functionalized component which had anode and cathode compartment to attract cfDNA; and real-time monitoring control, namely, a three-electrode prototype CRISPR-Cas9 MN system.

**Fig. 1:**
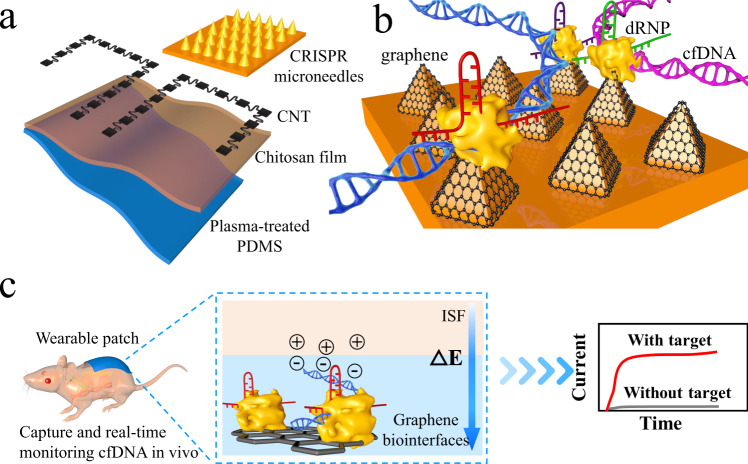
Schematics of CRISPR-Cas9 activated graphene biointerfaces for extraction and real-time in vivo monitoring of universal cfDNA. **a** Workflow of the wearable patch fabrication. **b** Scheme showing CRISPR microneedles integrated with the dCas9 enzyme and a sequence-specific sgRNA (denoted as dRNP) immobilized on a carboxyl graphene surface. **c** Real-time monitoring of the enriched cfDNA based on reverse iontophoresis and CRISPR-Cas9 activated graphene biointerfaces. The specific recognition of cfDNA to dRNP regulates the electrochemical characteristics and potential difference of the graphene layer, generating electrical signal output.

As shown in Fig. 1a, to achieve real-time monitoring of target DNA, the proposed wearable platform is composed of a spray-printed functional flexible patch and three-electrode conductive MNs. First, the surface of the PDMS membrane was treated with plasma to increase the hydrophilicity of the membrane. Then, a hydrophilic membrane was fabricated on the PDMS membrane via drop-casting of 1% chitosan solution. Due to the soft characteristics and weak surface adhesion of PDMS, the percolating microstructure would be deformed out of the interface during bending, stretching, and twisting^24^. Inspired by these properties, CNTs were deposited on the modified PDMS film by inkjet printing using a spray gun (0.17 MPa, 300 μm diameter) in this study^12^. The printed CNT pattern acted as a reverse iontophoresis compartment, separating negatively charged compounds (e.g., nucleic acids or ascorbate). Finally, a conductive CRISPR microneedle array as the working electrode was attached to the anode side of the CNT pattern. The CRISPR MN showed three functions during real-time detection: (I) insertion into the epidermis to isolate and concentrate target DNA; (II) CRISPR gene editing specifically performed by Cas9/sgRNA immobilized on the surface of the CRISPR MNs; and (III) the formation of a three-electrode system to record electrical signals.

Figure 1b shows a scheme of CRISPR MNs construction. In this CRISPR-Cas system, we used a catalytically inactivated Cas9 enzyme (dCas9) to form Cas9/sgRNA, denoted as dRNP^25^. Although both nuclease domains (RuvC and HNH) are deactivated in dCas9, the dRNP retain the ability to bind specifically to target DNA^13,26,27^. Immobilized dRNP can scan the entire DNA sequence under the guidance of sgRNA, where a 20-nt specific sequence matches the target DNA^14^. Once matched, dRNP can unwind the double-stranded helix and specifically bind with target DNA directly upstream of the 5′-NGG protospacer adjacent motif (PAM). The real-time monitoring capability of the wearable patch may come from two aspects: (I) dRNP of CRISPR-Cas9 as a driving force continuously searched and recognized target DNA; and (II) graphene biointerfaces on MNs provided highly efficient charged compound interactions and electron transport. In Fig. 1c, hybridization of dRNP on the surface of graphene with CRISPR gene editing targets not only altered the conductivity of the graphene interface channel but also resulted in counterion accumulation. Therefore, an ion-permeable layer was generated on the graphene surface to maintain charge neutrality. The difference in ion concentration between the bulk solution and the ion-permeable layer produced the Donnan potential^28^. Hence, the recorded output electrical signals can reflect the real-time recognition of the target cfDNA, and the theory and corresponding verification are deduced in the Supplementary Information (Supplementary Note 1).

### Validation and affinity of dRNP to target DNA

To validate the feasibility of the CRISPR wearable system, we first tested the CRISPR-Cas9 reaction for EBV cfDNA gene editing in solution. In earlier studies^22,23^, it was reported that the infection of Epstein-Barr virus (EBV) is closely related with various diseases, including acute virus abscesses, infectious mononucleosis, post-transplant lymphoproliferative disorder, and nasopharyngeal carcinoma. EBV cfDNA was released by cell apoptosis and necrosis in patients with distant metastasis or localized diseases. Therefore, it is worthwhile to monitor circulating EBV cfDNA real time in a minimally invasive and specific manner.

**Fig. 2:**
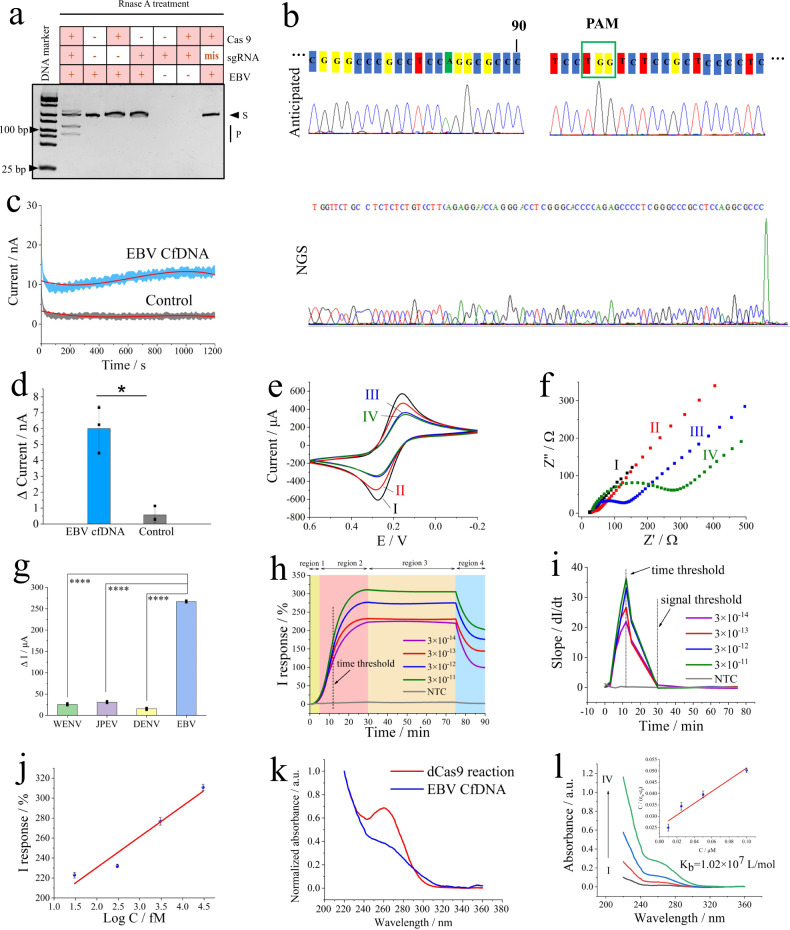
Validation of the CRISPR system and off-wearable strategy targeting EBV cfDNA on a solid commercial microelectrode. **a** In vitro cleavage ability of dRNP validated by polyacrylamide gel electrophoresis (PAGE), S and P refer to the sample and product, respectively. **b** Next-generation sequencing of CRISPR-Cas9 gene editing in EBV cfDNA. **c** CRISPR-Cas9 system representative real-time *i*–*t* curve raw data for detecting EBV cfDNA targets (sample interval 0.1 s, sampling time 1200 s); the red line represents the fitting curve (polynomial order = 3). **d** The current signal output of the CRISPR-Cas9 system in the presence of EBV cfDNA, analyzed by two-way ANOVA, *p* value = 0.038, *n* = 3 independent experiments, data presented as mean values ± standard deviation (SD). **e** CV plots and **f** EIS spectra of the microelectrode under different conditions. I, II, III, and IV refer to the bare microelectrode, graphene-modified microelectrode, CRISPR microelectrode, and CRISPR microelectrode targeting 2 × 10^−10 ^M EBV cfDNA, respectively, using 0.05 M [Fe(CN)_6_]^3−/4−^ as the probe. **g** Specificity of the CRISPR-based microelectrode; 100 times of interferences showed a slight influence, 0.05 M [Fe(CN)_6_]^3−/4−^ as the probe, *p < 0.05, **p < 0.01, ***p < 0.001, ****p < 0.0001, analyzed by two-way ANOVA, *p* value of 0.0000025, 0.0000018, 0.000017 for WENV, JPEV, DENV respectively, data presented as mean values ± SD, n = 3 independent experiments. **h** Real-time CRISPR-based microelectrode I response targeting variable concentrations of EBV cfDNA. Regions 1, 2, 3, and 4 refer to the phase before the time threshold, the phase after the time threshold, the stable period, and the rinsing step, respectively. **i** Slope of the plots from (**h**). **j** Calibration curve of the real-time I response from (**h**), data presented as mean values ± SD, *n* = 3 independent experiments. **k** The interaction of dRNP and EBV cfDNA. **l** UV-vis absorption changes; inset: calibration curve, dRNP with EBV cfDNA; I, II, III, and IV: R_0.1_, R_0.25_, R_0.5_, and R_1.0_, respectively, data presented as mean values ± SD, *n* = 3 replicated measurements.

From the genotyping data in Fig. 2a, b, two new bands in lane 1 were observed due to CRISPR gene editing, which contained Cas9, sgRNA, and EBV cfDNA. In addition, it was elucidated that the CRISPR reaction did not occur with mismatched sgRNA or sgRNA-free sequences. Accordingly, sgRNA plays an important role in the CRISPR-Cas system^14^. To this end, optimized experiments for sgRNA screening were performed in this study (Supplementary Fig. 2). The effect of the selected sgRNA on triggering CRISPR-Cas9 was verified in a concentration-independent manner, as shown in Supplementary Fig. 2. According to region of interest (ROI) analysis of the PAGE gel results, the average ROI value of the CRISPR product bands gradually increased, while that of EBV cfDNA decreased (Supplementary Fig. 2).

Then, we used a commercial solid microelectrode for EBV cfDNA target CRISPR gene editing on a skin chip (37 °C, pH 7.4). Figure 2c shows the original *i*–*t* curve data in response to 10^9^ copies/μL EBV cfDNA. Compared with that of the control group, the fitting curve of EBV cfDNA was stable within 200 s and gradually increased after 400 s. The results showed that the current output signal comes from the directional recognition and binding of the target by the dRNP complex. In Fig. 2d, there was a significant difference in the current between the positive and control groups, which was related to the appearance of the Donnan potential. These results might primarily demonstrate the proposed mechanism by which the dRNP compound immobilized on microneedles plays an important role in real-time online capture and monitoring of target DNA.

In this study, a CRISPR-Cas9 driving strategy was designed for wearable patches to monitor the target cfDNA in real time. Therefore, the most important aspect is to ensure that dRNP has the ability to recognize and detect target cfDNA. For this purpose, we conducted experiments on a solid-state microelectrode (schematic in Supplementary Fig. 3). The targeting dRNP was modified on the surface of the microelectrode by a method similar to that used to prepare conductive microneedles. The CV and EIS characterization results using 0.05 M [Fe(CN)_6_]^3−/4−^ as a probe confirmed the successful fabrication of the CRISPR microelectrode (Fig. 2e, f). In comparison to that of the bare microelectrode, the peak redox current of the modified microelectrode was decreased because the repulsive force between the probe and CRISPSR-Cas9 sensitive film hindered interface electron transfer.

To evaluate the specificity of CRISPSR-Cas9, conserved sequences of West Nile virus (WENV, GenBank No. M12294.2), Japanese encephalitis virus (JPEV, GenBank No. NC001437.1), and dengue virus (DENV, GenBank No. AF326573.1) cloned into the PUC57 plasmid were chosen for interference (1 × 10^−8^ M). As shown in Fig. 2g, compared with the detection of 1 × 10^−10^ M EBV cfDNA, the current intensities of the interference group did not change obviously and had higher significance.

To explore the quantitative analysis and real-time ability of this method, the CRISPR microelectrode was applied to test variable concentrations of EBV cfDNA. According to reference^20^, we used Eq. (1) as the unit of this real-time monitoring, where I response reflected the change between I_t_ (measurement after incubation) and I_b_ (calibration background before measurement).

1 $${{{{{\rm{I\; response}}}}}}( \% )=\frac{{{{{{{\rm{I}}}}}}}_{{{{{{\rm{t}}}}}}}-{{{{{{\rm{I}}}}}}}_{{{{{{\rm{b}}}}}}}}{{{{{{{\rm{I}}}}}}}_{{{{{{\rm{b}}}}}}}}\times 100 \%$$

In Fig. 2h, the real-time monitoring plots could be divided into four regions: (I) region 1 (*t* < 5 min), where the signal did not increase significantly and was basically in a fluctuating state; (II) region 2 (5 min < *t* < 30 min), where the signal response of the positive sample increased drastically, but that of the NTC group did not change; (III) region 3 (*t* > 30 min), where the signal of the positive groups tended to be stable, which might indicate that the CRISPR reaction on the interface reached adsorption equilibrium under reverse iontophoresis; and (IV) region 4 (simulating drug treatment, TE buffer under stirring, pH 8.0, 15 min, 37 °C), where some EBV cfDNA on the interface was eluted, and the signal response value decreased. However, the NTC group did not show a corresponding signal response to these four processes. Similar to nucleic acid amplification (e.g., PCR)^29,30^, we hypothesized that there might be a defined time threshold for this protocol. The derivative of the real-time I response was obtained in Fig. 2i, that is, dI/dt and CRISPR reaction time. The time threshold of this experiment was defined as ~12 min.

To test whether this assay was quantitative, we defined a signal threshold for varying concentrations of EBV cfDNA. According to the derivative curve, we found that there was no significant change after 30 min, which was chosen as the signal limit. In Fig. 2j, within the signal threshold, a linear relationship was observed between the change in the I response and EBV cfDNA concentration (fM, C) in the range of 30–30,000 fM following the equation ΔI response (%) = 30.8316·lgC + 168.8204 (*R* = 0.9736), with a detection limit of 1.1 fM (LOD = 3δ_b_/K). In addition, the end-point method and EIS dynamic curves further demonstrated the feasibility of this strategy, as shown in the Supplementary Figs. 4–7. In particular, this kind of label-free biosensing strategy using hybrid nanomaterials with high carrier mobility, such as graphene^3^ or CNTs^7^, can mitigate charge shielding effects and sensitivity limitations. Herein, dRNP immobilized on graphene biointerfaces could be used to trigger the event of target DNA detection without reagents or bulky equipment.

The above results primarily illustrated that dRNP on the surface of the microelectrode can recognize and bind target DNA. We were also interested in the binding constant between dRNP and EBV cfDNA; therefore, UV-vis spectrophotometry was employed to verify the interaction between the two^11^. As seen from the data in Fig. 2k, l, the binding constant of *K*_b_ = 1.02 × 10^7^ L/mol indicated that there was a good interaction between dRNP and EBV cfDNA. These results suggested that CRISPR-Cas9 system can be employed in the subsequent microneedle array to achieve real-time monitoring.

### Characterization and evaluation of the CRISPR wearable patch

**Fig. 3:**
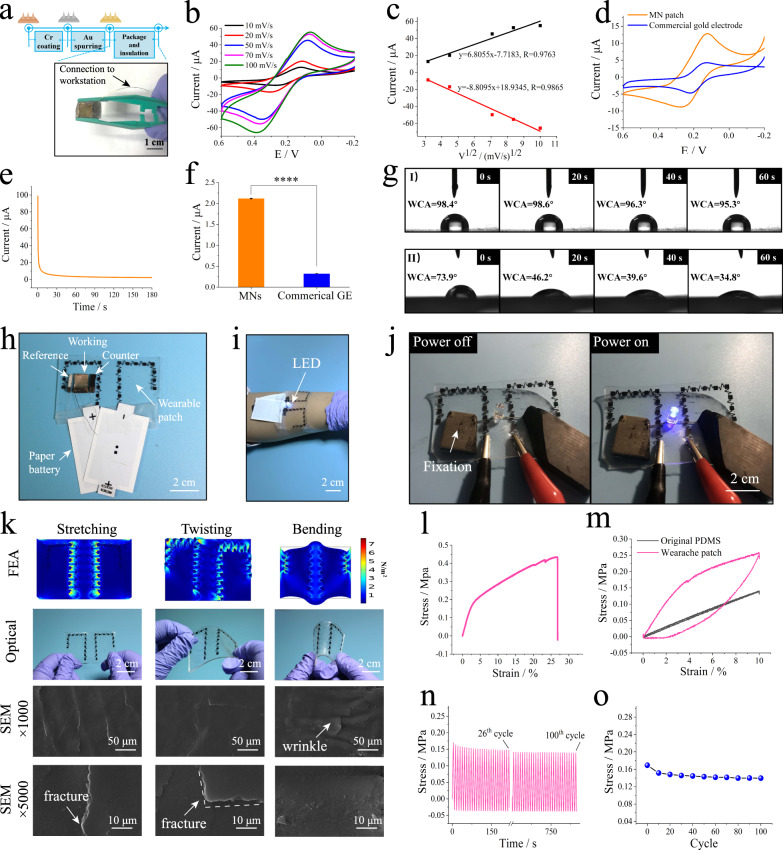
Fabrication, electrochemical and mechanical properties of the CRISPR wearable patch. **a** Schematic illustration of the conductive MNs. **b** CV plots of the as-fabricated conductive MNs under different scanning rates, using a 1 mM [Fe(CN)_6_]^3−/4−^ probe; quiet time, 2 s; sensitivity (A/V), 1 × 10^−4 ^A/V. **c** The relationship between the square root of the scanning rate and the corresponding peak current using a 1 mM [Fe(CN)_6_]^3−/4−^ probe; quiet time, 2 s; sensitivity (A/V), 1 × 10^−4 ^A/V. **d** CV plots of the conductive MNs and commercial gold electrode using a 1 mM [Fe(CN)_6_]^3−/4−^ probe; quiet time, 2 s; sensitivity (A/V), 1 × 10^−4 ^A/V. **e** Real-time *i*–*t* curve recorded by the conductive MNs in PBS buffer (0.01 M, pH 7.4); quiet time, 0 s; sensitivity (A/V), 1 × 10^−3 ^A/V. **f** The real-time current of the conductive MNs and commercial gold electrode in PBS buffer (0.01 M, pH 7.4); *p < 0.05, **p < 0.01, ***p < 0.001, ****p < 0.0001, analyzed by two-way ANOVA, *p* value of 0.0000094, quiet time, 0 s; sensitivity (A/V), 1 × 10^−3 ^A/V. **g** Contact angle testing of water droplets on the surface of the (I) original PDMS film and (II) hydrophilic-treated PDMS film. **h** Photograph of the CRISPR wearable patch based on reverse iontophoresis and three-electrode MNs. **i** The printed wearable patch mounted on skin. **j** A blue LED powered by the wearable patch with a voltage of 6 V. **k** Finite elemental analysis, optical photographs, and SEM of the wearable device under different mechanical distortions, including stretching, twisting, and bending; the results obtained from three independent repeated experiments. **l** Strain versus stress curve for the wearable patch. **m** A single stretch-release cycle with 10% strain for two different membranes. **n** Stress variation of the wearable patch in a 100-cycle test with a strain of 4%. **o** Stress changes of the wearable patch every ten cycles.

In this study, we fabricated CRISPR MNs using a series of methods, including phase 1 of metalation and phase 2 of CRISPR system functionalization. The detailed preparation and optimization procedures are discussed in the sections “Preparation of conductive microneedles” and “Functionalization, characterization of CRISPR micro-electrode and CRISPR microneedles”. From the results of Supplementary Fig. 8, we found that the rigidity and modulus of the microneedles were closely relative to its shapes and inertial distance. In addition, to test whether the graphene biointerfaces on the MN surface were rigid enough to perform the CRISPR reaction, we compared the graphene nanoflakes/chitosan membranes under different conditions by scanning electron microscopy (SEM) (Supplementary Fig. 9). From the results of atomic force microscopy (AFM) and conductive testing (Supplementary Figs. 20–22 and Supplementary Note 7), it deduced that the graphene nanoflakes/chitosan and dCas9 were successfully modified on the microneedle surface via drop-casting method and covalent bond, respectively. Figure 3a showed an off-the-shelf MNs that can be used directly for CRISPR-Cas9 decoration and wearable application. As shown in Supplementary Table 1, conductive MNs have been increasingly considered a promising tool for continuously monitoring from small molecules to biological macromolecules (e.g., RNA, DNA, protein), while it is still challenging to realize sample extraction and detection of nucleic acids simultaneously. In our research, reverse iontophoresis was used for preliminary enrichment and separation of the samples, which is an effective candidate for microneedles extraction function^12,31^. On this basis, real-time monitoring was performed by conductive MNs.

To test the quality of the prepared MNs, cyclic voltammetry (CV) was performed using [Fe(CN)_6_]^3−/4−^ as a probe, as shown in Fig. 3b, c. From the data, it was observed that the area of the CV plot increased as the scanning rate increased. Two linear relationships between the scanning rate and redox peak current were obtained. The above results implied that the well-defined conductivity and mass transfer of the MNs were subject to a diffusion-limited mode^32^. Due to the high specific surface area, the prepared MNs outperformed a commercial gold electrode (GE, diameter of 2 mm) at a peak current of 1 mM [Fe(CN)_6_]^3−/4−^ probe (Fig. 3d). One of the concerns was whether the MNs could be utilized for real-time *i*–*t* measurement. Therefore, we compared MNs with commercial GE in PBS buffer (0.01 M, pH 7.4) in Fig. 3e, f for real-time recording. Compared with commercial GE, MNs had reliable electrochemical performance and amplified the electrical signal by 6.5 times. In addition, the stability of MNs was investigated by CV measurements in different periods of 3 days, with an RSD of 9.04% (*n* = 9). The biosafety and biocompatibility of the MNs were also investigated in Supplementary Information (Supplementary Fig. 16 and Note 3).

To construct the wearable patch, polydimethylsiloxane (PDMS, Sylgard 184, Dow Corning) was chosen as a candidate substrate due to its elastic and stretchable properties. However, it is generally believed that the interface of PDMS is somewhat hydrophobic, which limits its application in wearable chem-biosensors^33^. One ideal method was to obtain the hydrophilic surface of PDMS by using stretchable and conductive nanomaterials, such as CNTs. Based on our previous report^12^, we first modified the PDMS surface primarily by plasma treatment and then drop-casted 1% chitosan solution. The wettability of PDMS was characterized via a water contact angle (WCA) meter. As shown in Fig. 3g, the droplets on the modified PDMS film changed significantly within 60 s (row II), while those on the surface of the original PDMS film changed little (row I). Through five-point fitting of the droplet distribution, the WCA of the modified PDMS film changed from 73.9° to 34.8°, and that of the original PDMS film changed from 98.4° to 95.3°. These results indicated that the surface wettability of PDMS had been effectively improved, which was probably due to the high permeability and good hydrophilicity of chitosan.

In Fig. 3h and i, demonstration of a skin-interfaced CRISPR wearable patch that integrated a reverse iontophoresis module and MNs biosensor for real-time tracking of target cfDNA was shown. As shown in Fig. 3j, to further test the practicability of the CNT printed wearable patch, a blue light-emitting diode (LED) was activated by the patterned conductive region. The external power supply was 6 V, which implied the good conductivity of the printed wearable material for subsequent experiments. The printed wearable patch exhibited stable electrical performance in the static (Supplementary Movie 1) or moving state (Supplementary Movie 2). The concept of representative wearable patches has been validated by a finite element analysis (FEA) simulation under different mechanical distortions, including stretching, twisting, and bending (Fig. 3k). The theoretical maximum modulus of the elastic wearable device at 16% stretch is ~0.07 MPa, which is comparable to human skin modulus^34^, indicating that it can be conformally mounted on the skin.

One ideal method to fabricate stretchable sensors has typically involved depositing CNTs on the surface of PDMS films^24,35^. It is commonly recognized that soft PDMS allows deformation of the percolating network microstructure during different mechanical distortions, which may lead to cracks on CNT membranes^36^. As presented in Fig. 3k, we further explored the morphology of CNT-printed PDMS using SEM to understand the relationship between the CNT percolating network and the deformation of the modified PDMS film. After stretching and twisting the substrate film, it was observed that the resulting fractures tended to be in the direction of deformation, resulting from uniaxial or biaxial distortions. The bending action induced wrinkles along the uniaxial direction. The results showed that CNTs deposited on the surface of PDMS were connected with each other, forming a percolating network, and the electron pathway was unblocked during the different deformation processes. The skin irritation of the CNT-printed wearable patch is one of the biosafety for medical device. To this end, piglet skin histological analysis and visual analog scale (VAS, score range from 0 to 10) for human volunteers were conducted to explore the biosafety of the iontophoretic wearable patch (Supplementary Fig. 15, Supplementary Note 2).

To verify the stretchability of the wearable patch, a series of mechanical property tests were carried out, as shown in Fig. 3l–o. The maximum elongation at break of the prepared patch reached 26.8% in the range of ~0.4 MPa. During a stretch-release test, hysteresis of the patch was clearly observed at 10% strain, which could be attributed to the multiple modification layers on the PDMS surface. Endurance tests confirmed that this wearable patch had good fatigue resistance, with a coefficient of variation (C.V.) of 17.6% in 100 cyclic strain tests. For stretchable electronic devices, the gauge factor (GF) is one of the most important parameters to evaluate the sensitivity of devices, as shown in Eq. (2) below^37^.

2 $${{{{{\rm{GF}}}}}}=\frac{\triangle {{\mbox{R/}}}{{{\mbox{R}}}}_{0}}{\varepsilon }$$

∆R/R_0_ and *ε* refer to the stress change and strain, respectively. The GF value of this patch reached 282.6 with a maximum strain of 26.8%. According to Euler-Bernoulli beam theory^38^, the bending resistance is proportional to the cube of the film thickness. Briefly, a thinner film is more flexible to mount the skin. Thus, the thinner the film is, the more elastic it is against the skin. Therefore, the surface modification of PDMS by chitosan with a high modulus result in low tensile properties but high sensitivity. For stretchable electronics, it is challenging to consider the effects of GF and strain simultaneously. The stretchable patch in this study demonstrated its reliability in the real world, even when compared to reported state-of-the-art flexible devices, such as polyurethane-PDMS nanomesh (GF = 46.3, strain ≈ 75%)^39^, nanofibril percolated PDMS (GF = 33, strain = 50%)^40^, and self-healable semiconducting polymer film (GF = 5.75 × 10^5^, strain = 100%)^41^.

**Fig. 4:**
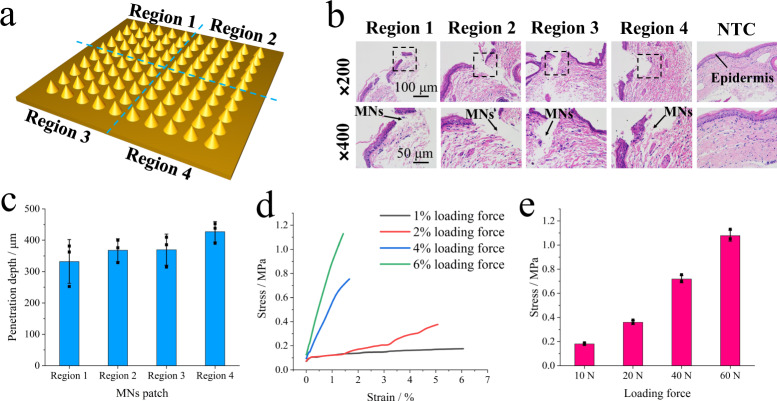
Mechanical properties of the microneedles. **a** The schematic for four regions of the MNs, each group of 25 needles. **b** Histological analysis of piglet skin after MNs administration, stained with HE, the black arrow referring to the direction of microneedles insertion, the black short dash box referring to the region of magnification photograph (×400). **c** The depth of MNs penetration into piglet skin, collected from four regions, data presented as mean values ± SD, *n* = 3 independent experiments. **d** The compression tests for the microneedle arrays with different loading forces, using 1 kN force sensor. **e** The evaluation of mechanical strength for the microneedle arrays, the maximum stress data collected from (**d**), data presented as mean values ± SD, *n* = 3 independent experiments.

Parallelly, penetration depth and mechanical strength of the graphene microneedles should be taken into consideration. The height of the graphene MNs was shown in Supplementary (height 600 ± 50 μm, microneedle tip 30 ± 10 μm, Supplementary Fig. 8d), which exerted an impact on the depth of penetration. To this aim, we sought to test the MN patch on piglet skin in vivo. In order to evaluate these needles, we divided the MN patch into four regions (Fig. 4a). From the results of Fig. 4b, c, histological analysis of hematoxylin and eosin (HE) was evident that the graphene MNs inserted into the piglet skin tissue (epidermis thickness of ~27 μm). And from four regions of histology section analysis results, the average length of microneedles tips inserted in the piglet tissue was quantified to be the range of 332.2–426.9 μm. In an early report^42^, it was confirmed that human epidermis thickness varied with different body sites, with the average of 40–50 μm. Thus, it leads us to believe that the height of the graphene MNs was capable to sample ISF. Then, compression tests were conducted under different loading forces via a force sensor (maximum of 1 kN) to study the mechanical strength of the graphene MNs. In Fig. 4d, e, as the loading force increased (1%, 2%, 4%, and 6%), the maximum stress of the graphene MNs increased, without obvious rupture or collapse. Under 6% loading force conditions, each needle stress completely exceeded the value of 43.1 kPa. Since the elastic deformation of the human skin can be as high as 15%, its modulus ranges from 10 to 200 kPa^43^. Namely, these results confirmed that the graphene MNs was able to penetrate epidermis and capture ISF biomarkers.

### In vitro extraction and real-time monitoring of cfDNA using a CRISPR MN patch

**Fig. 5:**
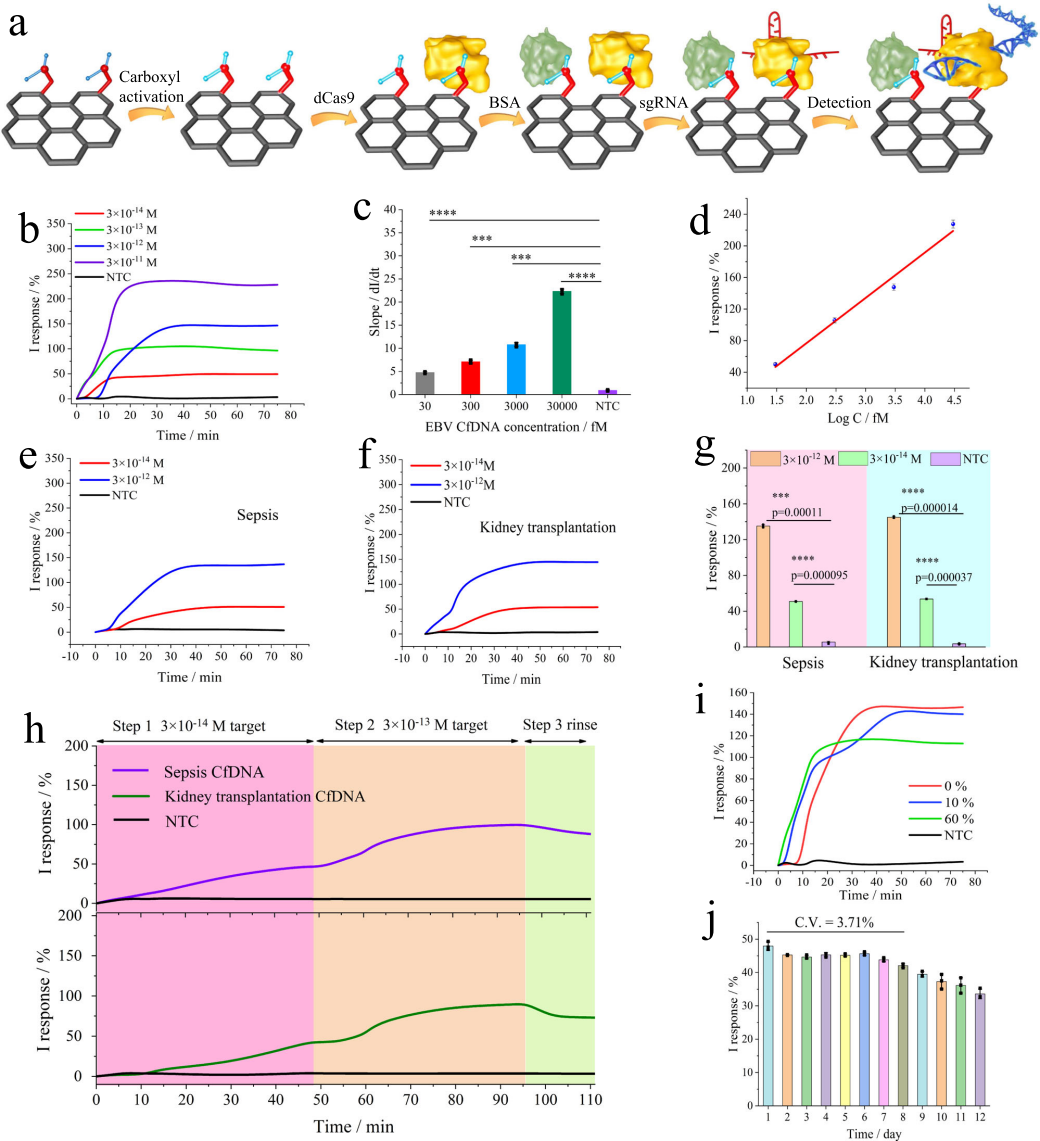
Analytical performance of CRISPR MN targeting different cfDNA in vitro. **a** Schematic of CRISPR MN preparation. **b** Real-time CRISPR MN I response targeting variable concentrations of EBV cfDNA, in the presence of the simulated ISF solution. **c** Slope values of different target detection, data collected from the curve peak of the real-time curve calculated by simple differentiation, the slope of EBV cfDNA target compared with no target control (NTC) using two-way ANOVA: *p < 0.05, **p < 0.01, ***p < 0.001, ****p < 0.0001, *p* value of 0.000092, 0.00038, 0.00027, 0.000096 for 30, 300, 3000, 30000 fM target DNA respectively, data presented as mean values ± SD, *n* = 3 independent experiments. **d** Standard calibration curve of the real-time I response from (**b**), data presented as mean values ± SD, *n* = 3 independent experiments. **e** Real-time CRISPR MN I response targeting variable concentrations of sepsis cfDNA, in the presence of the simulated ISF solution. **f** Real-time CRISPR MN I response targeting variable concentrations of kidney transplantation cfDNA, in the presence of the simulated ISF solution. **g** Signal response for sepsis cfDNA and kidney transplantation cfDNA in vitro including 3 × 10^−12 ^M, 3 × 10^−14 ^M, NTC, using two-way ANOVA: *p < 0.05, **p < 0.01, ***p < 0.001, ****p < 0.0001, data presented as mean values ± SD, *n* = 3 independent experiments. **h** Dynamic change of sepsis cfDNA and kidney transplantation cfDNA recording by CRISPR MN platform, steps 1–3 referring to real-time monitoring of 3 × 10^−14 ^M target cfDNA, 3 × 10^−13 ^M target cfDNA, rinsed by TE buffer (37 °C, pH 8.0). **i** Anti-interference ability of the CRISPR MN for 3 × 10^−12 ^M EBV cfDNA in the presence of various concentrations of fetal bovine serum. **j** The stable sensitivity of the CRISPR MN in vitro for 12 days, the CRISPR MN incubated in stimulated tissue and monitoring 3 × 10^−14 ^M target cfDNA on a skin chip under reverse iontophoresis (10 V), data presented as mean values ± SD, *n* = 3 independent experiments.

The ultimate goal of the proposed real-time method was to realize proof-of-concept recognition of cfDNA on wearable MNs. It is essential to determine the anti-interference and sensitivity of this system. Thus, based on our previous report^12^, we used a skin chip to simulate human skin (37 °C, 10 V of reverse iontophoresis) as an in vitro real-time monitoring setup for the performance evaluation. The skin chip consisting of three layers, including the epidermis, dermis, and endothelium, were developed. And detailed design, fabrication, and characterization of are discussed in the Supplementary Information (Supplementary Fig. 18 and Note 5). As mentioned above, original conductive MNs were obtained for subsequent decorations, as shown in Fig. 5a. Importantly, dCas9 was covalently immobilized, allowing the nuclease to bind tightly to the graphene surface.

The LOD of the microneedle patch not only plays an important role in real-world applications, but also ensures the sensitivity as well as detection accuracy, due to the low-abundance of cfDNA in ISF. Thus, we also investigated the real-time monitoring, sensitivity, and detection limit of the CRISPR MNs, as presented in Fig. 5b. Under reverse iontophoresis on the skin chip, CRISPR MNs were applied for EBV cfDNA detection, in the presence of the interferences that might exist in ISF and hinder the electrochemical detection (Supplementary Fig. 17 and Note 4). In contrast to the NTC group, EBV cfDNA was recognized and bound by dRNP on the CRISPR MNs surface in the four positive groups, producing significant signal output (analyzed by two-way ANOVA). As the concentration of EBV cfDNA increased, the relative I response increased, which corresponded to the CRISPR microelectrode. From the result of *i*–*t* curves, we found that the signal tended to be stable within ~30 min, illustrating that the total monitoring time of 75 min is sufficient.

As seen from the results of derivative calculation results in Supplementary Fig. 12, the positive groups had an obvious time threshold when compared with the NTC group. Interestingly, the time threshold increased as the concentration of the target DNA increased. This result can be attributed to the following reasons: (I) CV testing showed that MNs were controlled by the diffusion-limited mode (Fig. 3b and c), which might have an impact on the time threshold of the CRISPR reaction; and (II) based on reported research where the saturation of I response was used to quantify the target DNA concentration^20^, we primarily speculated that this kind of non-amplified detection method without a cycle reaction could not quantify the target concentration by the time threshold only. (III) according to the reports^21^, the reaction rate of cas enzyme-based system might be approximate Michaelis-Menten enzyme kinetics.

To determine whether the target DNA signal from Fig. 5b was indeed truly positive, we compared the slopes calculated by simple differentiation method for different EBV cfDNA target concentrations, ranging from 3 × 10^−11^ M to 3 × 10^−14^ M. As shown in Fig. 5c, the result confirmed that there is a significant difference between the NTC group and the 3 × 10^−14^ M EBV cfDNA. The calculated slopes are proportional to the concentration of target EBV cfDNA. In Fig. 5d, a linear relationship was observed between the change in the I response and EBV cfDNA concentration (fM, C) in the range of 30–30,000 fM following the equation ΔI response (%) = 57.4·lgC-38.1 (R = 0.9916). Thus, it could lead us to confirm that the sensitivity of the CRISPR MNs was 3 × 10^−14^ M in the presence of interferences, with a detection limit of 1.2 fM (3δ_b_/K).

As a universal method for different applications, we further applied this platform for the longitudinal monitoring of other two target cfDNAs, including sepsis-associated cfDNA and kidney transplantation-associated cfDNA. First, sepsis is a systemic immune dysregulated response to host infection, leading to high morbidity and mortality in intensive care (ICU) patients. Patients with severe sepsis require the continuous real-time monitoring of physiological and biochemical indexes during the prolonged ICU treatment^44,45^. Accordingly, recent studies demonstrated that cfDNA is not only a prognostic, predictive biomarker of sepsis, but also contributes to the duration of the inflammatory response via toll-like receptors activation in immune cells^44,45^. Second, monitoring of allograft rejection is critical for the long-term survival of organ transplant recipients^46^. cfDNA from donor organs was detected in the recipient’s circulation after organ transplantation^47,48^, which may be related to cellular damage in the graft. Therefore, these cfDNAs from transplants can be used as a minimally invasive or non-invasive way to assess transplant rejection in recipients. According to the report of Dennis Lo’s research^48,49^, in woman with male kidney donor, the SRY gene on the Y chromosome was used as a biomarker for donor-derived cfDNA (~130 bp).

Thus, we developed CRISPR MN for the direct capture and real-time monitoring of sepsis and kidney transplantation-associated cfDNAs. The optimized sgRNA for sepsis and kidney transplantation cfDNA was shown in Supplementary Fig. 23. As presented in Fig. 5e, f, under reverse iontophoresis on the skin chip, CRISPR MN were applied for longitudinal cfDNA monitoring. As the concentration of target cfDNA increased, the relative I response increased, and time threshold decreased. In Fig. 5g, contrast to the NTC group, sepsis as well as kidney transplantation cfDNA was recognized and bound by dRNP on the CRISPR MN surface in the positive groups, producing significant signal output (analyzed by two-way ANOVA). We also further investigated the signal dynamic change of sepsis and kidney transplantation cfDNA via standard skin chip, as shown in Fig. 5h, respectively. As the concentration of target cfDNA increased, the signal increased, indicating the CRISPR MN platform had the ability to monitor different concentrations of target cfDNAs. As above-mentioned different applications, we believe that this established platform is readily available for longitudinal monitoring of target cfDNA in complicated matrices.

The anti-interference of the CRISPR MNs was tested for the detection of 3 × 10^−12 ^M EBV cfDNA with different concentrations of fetal bovine serum (FBS) and control samples, including 0%, 10 and 60% FBS. The signal was recorded by *i*–*t* curve, as shown in Fig. 5i. The CRISPR MNs generated a stable and well-defined current response with a relative standard deviation (RSD) of 2.49% under the interference of 10% FBS when compared to 0% FBS interference. Moreover, we observed that 60% FBS had an influence on the CRISPR MNs, and the RSD was 20.95%, but it still showed an “S” curve within 75 min. This capability could allow CRISPR MNs to be used for wearables in the real world.

Furthermore, the long-term monitoring without loss of device sensitivity is one of the most important parameters. Thus, the device was retained in the stimulated tissue in vitro (2% agarose gel) for the long-term stable sensitivity investigation. Herein, in order to evaluate the long-term stable sensitivity of the CRISPR MN, signal response and time threshold were utilized for each CRISPR MN, which was obtained from the monitoring of minimum detection limit cfDNA concentration (3 × 10^−14^ M). From the results of in vitro (Fig. 5j, Supplementary Fig. 24), it showed that the CRISPR MN for target cfDNA still maintained good signal response and time threshold in the first 8 days with a C.V. of 3.71%. And a good and stable signal response and time threshold were demonstrated during the 12-day in vitro monitoring of the cfDNA (3 × 10^−14^ M). In Supplementary Fig. 24, the S_t_ value (defined as signal platform threshold) and T_t_ value (defined as the time threshold corresponding to the maximum of signal response curve derivatives) were relatively stable during the first 6 days. After 6 days, although the shapes of the differential I response curves changed (that is T_t_ value changing), the S_t_ value changes little. Since the qualitative capability of the system largely depends on the S_t_ value, it can be elucidated that this system maintains a stable qualitative capability over 12 days. These results demonstrated that the sensitivity of CRISPR MN was stable in vitro for 12 days and would be suitable applied in longtime monitoring of related disease, especially used during the intensive care unit treatment.

In addition, CRISPR MN reproducibility was further evaluated by measuring C.V. parameter without initial calibration (Supplementary Fig. 25 and Note 8). The results indicated a C.V. parameter of 9.34%, with no significance (*P* value = 0.33). It was demonstrated that the CRISPR MNs were reliable for real-time monitoring of target cfDNA.

Based on the aforementioned experimental results and previous reports, the real-time monitoring capacity of this CRISPR MN patch might be attributed to synergetic effects: (I) graphene, due to its excellent electrical sensitivity to charged molecule interactions on its surface, has found great applications in flexible and scalable electronic devices^50^. This material acts as a channel between MNs and the epidermal microenvironment and is an ideal candidate to produce Donnan potential (Supplementary Note 1). (II) Programmable dRNP, which acted as the driving force, could automatically search the entire gene sequence of the nucleic acid in the sample without amplification until it matched the target sequence. Importantly, it exhibited high spatiotemporal resolution in short-lived off-target binding events (average <1 s)^51^.

Table 1 summarized some state-of-the-art amplification-free CRISPR methods for analysis targets. As shown, an unamplified detection strategy has been considered as a universal tool for molecular diagnosis since programmable sgRNA or CrRNA can be designed for different genomic samples. However, compared with HUDSON-SHERLOCK^15,16^ or DETECTR^17^ (detection limit down to aM levels), these reported amplification-free methods (mostly ranging from pM to fM levels) without PCR or other isothermal nucleic acid amplifications have yet to exhibit considerable sensitivity for low-abundance biomolecule detection. In this study, our proposed CRISPR wearable device combining CRISPR MN with stretchable electronics showed potential advantages for portable, miniaturized, and wearable point-of-care testing.

**Table 1:** Comparison of representative amplification-free CRISPR-Cas strategy.

Method	Target	LOD	Time	Equipment	Ref.
Combined CRISPR-Cas13a device	SARS-CoV-2 RNA	100 copies/μL	~30 min	Smart mobile phone microscopy	^21^
CRISPR-chip	Bfp-transfected HEK293T cells	1.7 fM	~15 min	Graphene-modified field-effect transistor	^20^
CRISPR microfluidic	MiR-19b and miR-20a	10 pM	~9 min	Electrochemical microfluidic biosensor	^18^
Electrochemical CRISPR biosensor	Transforming growth factor β1	0.2 nM	~60 min	Aptamer-based electrode	^19^
CRISPR-Cas12a Sensors based on functional DNA activator	ATP and Na^+^	0.21 μM for ATP; 0.1 mM for Na^+^	~40 min	Microcentrifuge tube	^60^
CRISPR-Cas9-mediated SERS assay	*S. aureus*, *A. baumannii*, and *K. pneumoniae*	14.1 fM for *S. aureus*; 9.7 fM for *A. baumannii*, and 8.1 fM for *K. pneumoniae*	~30 min	Microcentrifuge tube	^61^
Tandem CRISPR nucleases of Cas13 and Csm6	SARS-CoV-2 RNA	30 copies/μL	~60 min	Fluorescence detector	^54^
Type III-A CRISPR-Cas systems	SARS-CoV-2 RNA	2000 copies/μL	30 min	Microcentrifuge tube	^62^
CRISPR-Cas13-based digital detection	SARS-CoV-2 N gene RNA	~5 fM	<5 min	Microchamber-array chip	^63^
Wearable CRISPR-Cas9 patch	EBV, Sepsis, and kidney transplantation cfDNA	1.1 fM	~30 min	Conductive microneedles	This work

### Demonstration of the CRISPR MN wearable system in vivo

**Fig. 6:**
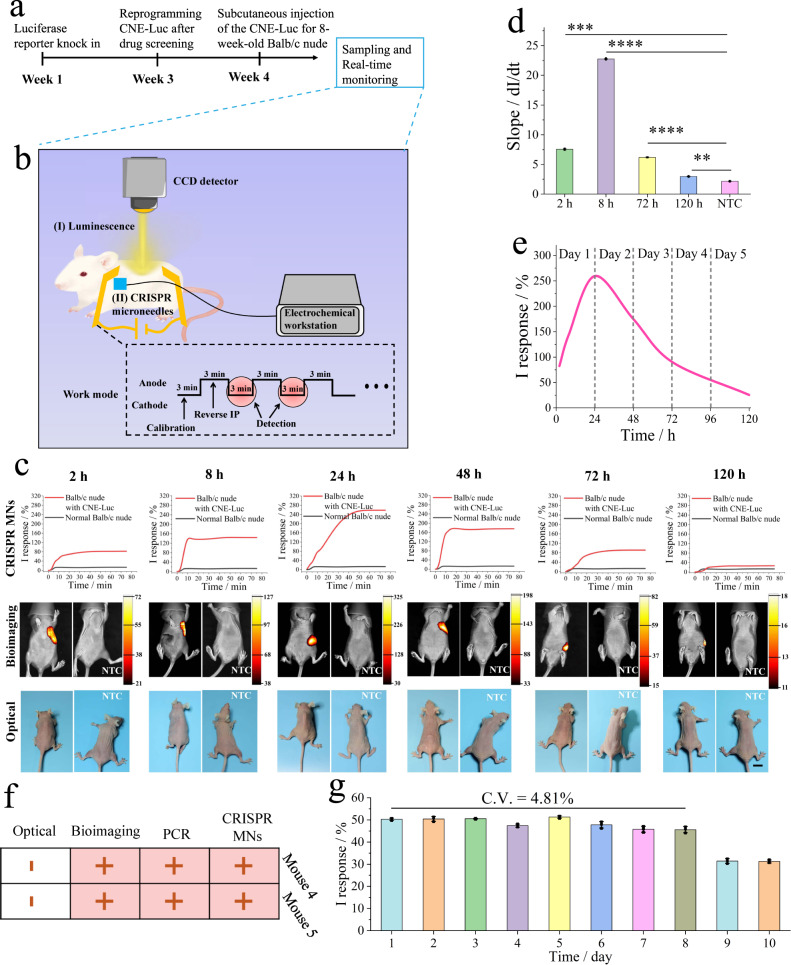
Demonstration of the CRISPR MN wearable system in mice. **a** Timeline of real-time monitoring in CNE-Luc-bearing mice. **b** Schematic illustration of in vivo real-time sampling and monitoring, including (I) chemiluminescence bioimaging and (II) CRISPR MN for CNE-Luc-bearing mice. The CRISPR MN system was calibrated in PBS (37 °C, 0.01 M, pH 7.4) for 3 min to eliminate sensor-to-sensor variation in electrical output. The red circle represented the detection time point. The skin stratum corneum of all BALB/c nude mice was cleaned by scrub cream, disinfection with 75% ethanol, and smearing with 100 μL TE buffer (pH 8.0). The region of interest on mouse skin was dried with cotton. Finally, the mice were placed on a heat plate during the real-time monitoring procedures. **c** Parallel trials on mice at different time points, including 2, 8, 24, 48, 72, and 120 h, scale bar: 1 cm. **d** Slope values collected from the curve peak of BALB/c nude mice real-time curve calculated by simple differentiation, including 2, 8, 72, 120 h, *p < 0.05, **p < 0.01, ***p < 0.001, ****p < 0.0001, as analyzed by two-way ANOVA, *p* value of 0.00011, 0.0000076, 0.000042, and 0.0045 for 2, 8, 72, and 120 h, respectively, data presented as mean values ± SD, *n* = 3 biologically independent animals. **e** In vivo dynamic change in EBV cfDNA levels in BALB/c nude mice detected by the CRISPR MN system during the first 5 days, the curve was fitted by spline curve mode, Origin 2018 software. **f** Four independent methods applied to 18-day CNE-Luc-bearing BALB/c nude mice demonstrated that the CRISPR MN wearable system was as accurate as the gold-standard PCR (blood sampling by a commercial kit) in terms of qualitative analysis. **g** The stable sensitivity of the CRISPR MN in vivo, the CRISPR MN laminated on BALB/c nude mice, then transferred to a skin chip for monitoring 3 × 10^−14 ^M target cfDNA under reverse iontophoresis (10 V), data presented as mean values ± SD, *n* = 3 independent experiments.

The experimental timeline of real-time monitoring of cfDNA in vivo based on reverse iontophoresis and CRISPR MN was demonstrated in Fig. 6a. Hereby in this study, to further verify the feasibility of the real-time online platform for in vivo cfDNA detection, EBV-mice model was applied for cfDNA monitoring in vivo. Thus, a luciferase reporter gene (Luc) was inserted into CNE cell lines and then subcutaneously inoculated into 8-week-old female BALB/c nude mice for subsequent experiments. Detailed cell and animal experiments were listed in the experimental section. Finally, the constructed CRISPR MN with corresponding sgRNA, integrated with reverse iontophoresis components (external voltage of 10 V) were employed in BALB/c nude mice.

To avoid signal crossover, this CRISPR MN platform applied an intermittent measurement, similar to the GlucoWatch® biographer (Cygnus, Inc., Redwood City, CA, USA)^52^, as shown in Fig. 6b. In brief, a voltage of 10 V was applied to extract the target for 3 min by reverse iontophoresis in the first step. Then, reverse iontophoresis was stopped, and the biosensor which remained to be laminated on the epidermis was engined for collecting electrochemical signal. The signal of this biosensor at the corresponding region was recorded for 3 min. These two steps were repeated to achieve real-time cfDNA monitoring.

As seen from the data in Fig. 6c, the signals of the CRISPR MN method (I response of 82.39%) and bioimaging method (maximum of 72 a.u.) were vividly identical 2 h after inoculating CNE-Luc, while optical imaging was ineffective for target screening at the early stage. Subsequently, at the 8-h time point (I response of 145.48%), the abundance of EBV cfDNA in mice monitored by our method was higher than that at the 2-h time point. At the same time, the bioimaging signal increased to a high value (maximum of 127 a.u.), consistent with CRISPR MNs. Then, at the 24-h time point (I response of 256.34%), the abundance of the target cfDNA in mice reached a peak in comparison of other time point groups, which was also consistent with the bioimaging signal (maximum of 325 a.u.). Subsequently, at the 72-h time point, our method could still monitor EBV cfDNA in real time (I response of 90.65%), and the bioimaging signal also decreased (maximum of 82 a.u.), possibly due to the heterogeneity of CNE-Luc cell lines during the formation of nasopharyngeal carcinoma. From the results of 120 h, although the bioimaging signal continuously decreased, it was still able to effectively distinguish the positive group (I response of 25.44%) and NTC group (I response of 11.20%). It could be concluded that EBV cfDNA was closely related to CNE-Luc cells. However, naked-eye visualization was unavailable for the first five days. This CRISPR MN platform can not only effectively monitor EBV cfDNA real time in vivo but also be used for the early screening of nasopharyngeal cancer tumors. To further test whether the real-time I response was indeed true, shown in Fig. 6d, we compared some curve slopes by differentiating the I response curves at various time points. This comparison confirmed that the slopes were proportional to the intensity of the biological imaging signals, and there was still a significant difference between the 120-h time point and NTC groups. During the first 5 days, the CRISPR MN wearable system was able to record dynamic changes in target DNA levels in BALB/c nude mice, showing the same trend as the bioimaging method (Fig. 6e). These results illustrated that this wearable system would be expected to be employed for real-time monitoring of target cfDNA.

Unlike traditional labs, a wearable device is exposed to an uncontrolled environment for a long time, which might pose a challenge in detection accuracy during continuous monitoring^2^. Therefore, we conducted four independent tests on 18-day CNE-Luc-bearing BALB/c nude mice to verify the accuracy of the CRISPR MNs (Fig. 6f, Figs. S10 and S11). For the CRISPR MN platform, the procedure is shown in Fig. 5a; for gold-standard PCR (kit provided by TIANGEN Co., Ltd., Beijing), the sampling blood was first treated by a commercial DNA extraction kit (provided by Sangon, Shanghai). Compared with PCR, CRISPR MNs ensured a reliable qualitative detection in mice, but their quantitative detection ability was not yet known.

In addition, we also study the long-term stability of the device in vivo on BALB/c nude mice. From the results of in vivo (Fig. 6g, Supplementary Fig. 26), it showed that the CRISPR MN was remained stable S_t_ and T_t_ in the first 8 days with a C.V. of 4.81%, where the signal response was in the range of 50.7% to 45.6% and time threshold was in the range from 11.1 to 14.8. It still exhibited obvious signal response as well as time threshold at the 10-day time point. To the best of our knowledge, such 10-day stability of the device for in vivo nucleic acid monitoring might be satisfactory so far, and can also meet the clinical needs for ICU treatment. Collectively, these results demonstrated that the dRNP with modified sgRNA were stable and able to tolerate the complicated matrices in vivo, contributing to long-term applications over 10 days.

## Discussion

In summary, based on the synergetic effect of CRISPR-Cas9 and graphene biointerfaces, this study proposed online wearable conductive MNs that perform highly effective extraction and real-time monitoring of target cfDNA. This CRISPR-Cas9 activated wearable patch could continuously monitor cell-free DNA targets in vivo, with a detection limit of 1.2 fM (3δ_b_/K) for CRISPR MN, with good electrochemical performance and stability for 10 days in vivo. Though the wearable microneedles could specifically recognize different target cfDNA via the programmable dRNP in this study, the established method is expected to be longitudinally applicable to various genomic sites of one target cfDNA. To this end, we also investigated the monitoring ability through four programmable sgRNA sequences (Supplementary Fig. 19 and Note 6). The results confirmed that the four sgRNA were highly specific to their target cfDNA. In this work, we mainly made use of the standard skin chip to demonstrate the application potential of our wearable system in other diseases, such as sepsis, kidney transplantation. Our following work will focus on the microneedle applications for sepsis and kidney transplantation in animal or human clinical models, to further study the basic mechanism of the dynamic change of nucleic acid biomarkers in ISF, and its relationship with the disease progression. As a sampling and real-time monitoring device for diagnosing patient’s disease, the proposed CRISPR-based microneedle might be applied for clinical surgery.

The real-time wearable device could overcome the “Black-Box” status of disease administration via combining on-demand capture and real-time feedback. More recently, in Dincer’s point of view^53^, capturing target cfDNA from an individual is just half the story, and the next-generation of wearable devices should have the ability to continuously monitor biomarkers in real-time. Indeed, real-time wearable devices have been emerging in the wearable commercial market, such as Freestyle Libre (Abbott Inc.) or G5 continuous glucose monitoring (Dexcom Inc.). Hence, real-time wearables could be more patient-friendly for disease-derived biomarker detection, such as cfDNA or proteins.

Importantly, swelling and dissolution kinetics of the MN substrate materials might influence sensing performance. To this aim, the MN was investigated on KM mice in vivo (Supplementary Fig. 14), with a maximum C.V. of <3.6% within 24 h. We further explored MNs using SU-8 2075 photoresist (MicroChem, USA) as the substrate material, whose swelling and dissolution kinetics were evaluated in PBS (pH 7.4, 37 °C, 20 min). Compared with the pristine SU-8 microneedles, there is little change in swelling and dissolution after incubation and dehydration, with a C.V.% of 0.11% and a C.V.% of 0.25%, respectively. We believe that the SU-8 substrate may offer the potential to fabricate MNs for real-time nucleic acids monitoring in the future.

However, two major challenges still remain in terms of wearable devices: (I) due to the instability of the immobilized bioreceptor, the interface between the device and the active sensitive film fluctuates during the dynamic deformation process. In addition, Joseph Wang et al. pointed out that the detection accuracy of wearables would be affected by the interface effect during continuous operation^2^. Unlike traditional laboratories, wearables are often exposed to harsh conditions that affect the bioactivity of immobilized receptors. Usage of a hydrogel or chitosan layer on the top to protect the immobilized bioreceptor may solve this issue. (II) Regarding sensitivity, probably due to the lack of preamplification, the current version of an amplification-free strategy could not meet the requirements of highly sensitive DNA detection. As shown in Table 1, the optimized sensitivity proposed by Liu et al. reached 30 copies/μL for SARS-CoV-2 RNA^54^, and that of our proposed assay was 1.1 fM for target cfDNA, which was still unavailable for low-abundance biomarker analysis in particular practical applications (single copies/μL). Therefore, subsequent studies, including those on multiple Cas proteins, ordered mesoporous nanomaterials, precalibration processes, and metallic microneedle patches, should further endeavor to improve interfacial receptor immobilization and sensitivity to achieve detection of single-copy DNA of interest.

## Methods

### Ethical statement

Animals were cared for and maintained under the Guidelines of Laboratory Animals of Fudan University. All experiments involving animals or animal products were approved by the Animal Ethics Committee of Fudan University, China (approval No. 2021JSCHEM-020). The human research was approved by Ethics Committee of Fudan University and complied with all relevant ethical regulations (IRB No. FE20037). Informed consent was given by human participants, and they were compensated.

### Synthesis of polymethyl vinyl ether-alt-maleic acid (PMVE/MA) hydrogel

The reagents were provided by Aladdin Corporation (Shanghai). Briefly, first, 10 g of PMVE/MA was dissolved in 60 mL of ddH_2_O and reflux-stirred for 24 h at 80 °C. After cooling, 6 g of polyethylene glycol (PEG, MW = 8000 Da) was added into the PMVE/MA solution for 12-h reflux-stirring at 28 °C.

### Preparation of conductive microneedles

We designed the conceptual microneedle scheme, and a metallic wafer with a negative surface pattern was processed by Wuxi Guorui Electronic Technology Co., Ltd. (Wuxi, China). The metallic wafer was used to replicate the PDMS mould shape of the microneedle array (12 × 12 microneedles, microneedle base diameter 300 ± 10 μm, microneedle height 600 ± 50 μm, microneedle tip 30 ± 10 μm, microneedle interval distance 300 μm). To obtain the hydrogel-based microneedle patch, three-step replication processes were conducted in this study. First, the metallic wafer was ultrasonically cleaned with ddH_2_O for 3 min and then dried in an oven (80 °C). Surface hydrophobic treatment was performed for metallic wafers for 5 min. PDMS (Sylgard 184 silicone elastomer, Dow Corning Inc.) was prepared at a weight ratio of base to curing agent of 10:1 and poured into the wafer. This metallic wafer mould was vacuumed (600 mmHg, 25 °C, 30 min) to remove air from the PDMS matrix and centrifuged for 30 min (1788.8 × *g*), which was repeated three times. Afterward, this metallic wafer was placed in an oven (80 °C, 4 h). The first replication of the PDMS microneedle patch was carefully peeled from the metallic wafer. Next, Ecoflex (smooth-on 0030) precursor mixture at a weight ratio of base to curing agent of 1:1 was poured carefully onto the PDMS master after surface hydrophobic treatment, which was vacuumed for 5 min to eliminate bubbles and cured at 80 °C for 4 h. Thus, the second replication was achieved. Finally, the synthesized PMVE/MA hydrogel (13 mL) in this study was poured into a holder where the Ecoflex master was fixed on the bottom. This holder was first placed in a vacuum oven (80 °C) for 7 h to ensure that the mixture solution filled the microwells. Then, the holder was transferred to an oven (90 °C, 24 h). Consequently, the holder was placed in a fume hood and peeled off immediately. A pristine hydrogel microneedle patch was obtained and stored in a desiccator at 20 °C when not in use.

For conductive MNs of working electrode, the fabrication steps are as followed: (i) the pristine MNs were first plasma-treated for 1 min. (ii) 100 μL 0.5 mg/mL Cr dispersion solution (Yingtai Metal Materials Corporation, Shandong, China) in 0.1% chitosan (Sinopharm Chemical Reagent Corporation, China) was drop-casted on the surface of MNs immediately. And it was placed in oven (60 °C, 30 min) to dry. (iii) A compact gold film was formed on its surface by Au spurring (10 mA, 600 s, EMS 150 ion sputtering instrument, USA). (iv) A gold wire (diameter of 200 μm) was attached to the contact area of the MNs by brushing carbon paste (SPI Supplies Co., USA) and placed in the oven (60 °C, 10 min). (v) It was coated a gold film by Au spurring (10 mA, 300 s, EMS 150 ion sputtering instrument, USA) to maintain a consistent surface. (vi) Insulation and package step were applied for the contact and non-conductive area (four sides and the back) by brushing Ecoflex (smooth-on 0030) precursor mixture at a weight ratio of base to curing agent of 1:1, and it was placed in oven (60 °C, 60 min).

For the conductive MNs of reference electrode, the fabrication processes include steps (i) to (vi). And the step (vii) is as followed: the MNs were pasted with Ag/AgCl ink (BAS Inc., Japan), cured at 80 °C for 30 min.

For the conductive MNs of counter electrode, the fabrication processes include steps (i) to (vi). And the step (vii) is as followed: the MNs were pasted with carbon paste (SPI Supplies Co., USA), cured at 60 °C for 10 min.

Thus, a three-electrode MNs system was ready for subsequence modification and application. The morphology and thickness of the MNs were validated by a stylus profiler (AlphaStep D-600, KLA-Tencor Corp.).

### Functionalization, characterization of CRISPR micro-electrode, and CRISPR microneedles

The metal micro-electrode (2 mm in diameter, gold electrode) was purchased from Gaoss Union Incorporation (Wuhan, China). For CRISPR micro-electrode construction, the steps are as followed: (i) 0.2 mg/mL graphene dispersed (nanoflakes, Sigma-Aldrich provided) in 0.1% chitosan was drop-casting on the surface and it was placed in oven (60 °C, 30 min). (ii) 10 μL 1-pyrenebutanoic acid (PBA, dissolved in N,N-Dimethylformamide, Aladdin Co., Shanghai) of 5 mM was drop-casted on this surface under 37 °C for 60 min. PBA was stacked on the graphene surface via π–π interaction. And the micro-electrode was washed by ddH_2_O. (iii) Accordingly^20,55^, carboxyl group of PBA was activated by N-(3-dimethylaminopropyl)-N′-ethylcarbodiimide (EDC, 4 mM) and hydrochloride/N-hydroxysuccinimide (NHS, 11 mM) solution (100 μL:100 μL in 100 μL 50 mM 2-morpholinoethanesulfonic acid buffer, all provided by Aladdin Co., Shanghai) for 60 min. (iv) The micro-electrode was incubated in 1 μM dCas9 (Tolo Biotechnology Corporation, Shanghai) for 60 min under 37 °C. (v) 1% BSA (bovine serum albumin, Sangon Corporation, Shanghai) blocked the non-specific active sites for 10 min under 37 °C. It was washed by 2 mM MgCl_2_ (Sangon Corporation, Shanghai). (vi) The micro-electrode was incubated in 10 μM sgRNA (GenScript Co., synthesized and provided, Nanjing, China) for 60 min under 37 °C, and washed by 2 mM MgCl_2_ for 1 min. Thus, a CRISPR micro-electrode was fabricated.

For CRISPR microneedles, the steps are as followed: (i) before construction, the conductive MNs were sterilized under UV light for 10 min and plasma-treated for 1 min. (ii) 100 μL 0.2 mg/mL carboxyl graphene (nanoflakes, XFNANO Co., Nanjing) dispersed in 0.1% chitosan was immediately drop-casted on MNs surface and placed in the oven for 30 min under 60 °C. (iii) Accordingly^20,55^, carboxyl group of graphene was activated by 100 μL mixed solution of EDC (4 mM): NHS (11 mM) with a volume of 200 μL:200 μL in 100 μL 50 mM 2-morpholinoethanesulfonic acid buffer (all provided by Aladdin Co., Shanghai) for 60 min under 37 °C. The remainder solution on the MNs surface was eliminated by pipetting and placed in the oven to dry (40 min, 37 °C). (iv) The MNs were incubated in 1 μM dCas9 (Tolo Biotechnology) for 60 min under 37 °C and dried in the oven (40 min, 37 °C). (v) 1% BSA blocked the non-specific active sites for 10 min under 37 °C and dried in the oven (40 min, 37 °C). (vi) The MNs were incubated in 10 μM sgRNA (GenScript Co., synthesized and provided, Nanjing, China) for 60 min under 37 °C and washed by 2 mM MgCl_2_ for 1 min. Finally, the MNs were dried in the oven (40 min, 37 °C). A CRISPR MNs was obtained. And it was stored in −20 °C refrigerator prior to utilization.

As for mechanical characterization, bare MN patch and graphene-modified MN patch were tested on Instron 5966 electronic universal testing machine (Instron, USA) respectively, using 1 kN force sensor (*n* = 3). The testing mode was compress testing under different loading force (2%, 4%, 6%). In order to investigate the swelling and dissolution of MN patch, healthy KM female mice (aged 4 weeks) were used as animal modes. The PMVE/MA/PEG bare MNs was modified with Cr film, gold film, successively. Then it was drop-casting graphene nanoflakes/chitosan solution. Finally, the as-prepared graphene-based MNs was laminated on healthy KM mice in different periods. Finally, differential pulse voltammetry testing was conducted to demonstrate the condition atop the MNs. The scanning potential range was set from −0.2 V to 0.6 V at a scan rate of 50 mV/s in a 0.05 M K_3_[Fe(CN)_6_]/K_4_[Fe(CN)_6_] solution that contained 0.50 M KCl.

As for the stability characterization, the MNs after metalation and functionalization, namely CRISPR-based MNs, were laminated on the BALB/c nude mouse in vivo that was cleaned by scrub cream (*n* = 3). And anesthesia was applied to the mice (2% avertin, 250 μL for each subject). However, the volume of 250 μL 2% avertin could only last for around 3 h. Thus, for the long-term stability testing in this study (24 h), anesthesia was applied to the mice every 3 h. Finally, it is transferred to skin chips to monitor targeted cfDNA at specific concentrations in real time.

For the evaluation of reproducibility, five different CRISPR microneedle sensors were prepared in different batches. The fabrication procedures were under the same condition as mentioned above. After incubation with 3.59 nM target EBV cfDNA under 37 °C for 60 min (the sample sequences checked by next-generation sequencing via Chromas version 2.3 software), five CRISPR microneedle sensors as working electrode were connected to the electrochemical workstation (CHI 1030, Shanghai, China) for collecting data (*i*–*t* curve). Current data was recorded in the simulated ISF (PBS, 0.01 M, pH 7.4); quiet time, 0 s; sensitivity (A/V), 1 × 10^−3^ A/V.

### Iontophoretic wearable patch construction

First, the crude PDMS precursor mixture at a weight of base to curing agent of 6 g:0.6 g was poured carefully onto the square plain petri dish master (diameter of 9 cm, Corning Corporation) after surface hydrophobic treatment, which was vacuumed for 5 min to eliminate bubbles and cured at 80 °C for 1 h. The crude PDMS thin film was peeled from the substrate. Meanwhile, the 1% chitosan solution was prepared (1 g chitosan in 100 mL of 2% acetic acid, stirring for 5 h) and centrifugated (894.4 × *g*, 30 min) to eliminate bubble. Second, the prepared PDMS thin film was plasma-treated for 4 min. The 1% chitosan solution of 2 mL was immediately casted on the surface of the PDMS thin film. And it was curing in the oven (80 °C, 1 h). Then, a chitosan-modified PDMS thin film was obtained. A general microfluidic mask with the desired pattern was gently placed on the surface of the modified thin film. A spray-gun (300 μm diameter, C100, Shibangde Corporation, Shanghai) spray-deposited the as-prepared CNT solution (XFNANO Co., Nanjing) to the modified PDMS surface through hollow region of the mask. The printed functional membrane was placed on a thermal plate to eliminate drops. Then, fabricated three-electrode microneedles were attached on the anode side of the thin film by 3 M tape. Finally, a multifunctional wearable device was obtained. All the mechanical testing was performed on Instron 5966 electronic universal testing machine (Instron, USA). Finite element analysis was conducted by COMSOL Multiphysics 5.3 software. The contact angle testing results were analyzed by JC 2000D software. The SEM was conducted on Zeiss Gemini SEM500 FESEM and VEGA 3 XMU (TESCAN Co., Czech).

### Typical CRISPR, PCR, and PAGE gel electrophoresis

A typical CRISPR reaction in a centrifugal tube was as follows. The pre-incubation solution comprised of 8.5 μL ddH_2_O, 3 μL buffer, 1.5 μL sgRNA (20 μM), 1 μL spCas9 (1 μM, Tolo Biotechnology Corporation, Shanghai), for 10 min, 25 °C. Then, 6 μL target cfDNA PCR product was added for CRISPR reaction (incubated for 90 min under 37 °C). Then, 1 μL Rnase A (5 mg/mL, Sangon Co., provided) was added to eliminate sgRNA for 30 min under 37 °C. Finally, it was incubated in 95 °C for 5 min to stop CRISPR as well as Rnase A reaction, releasing binding gene-edited EBV cfDNA^56,57^.

Conserved nucleic acid fragments of the EBV BamHI-W region were screened (GenBank No. A10072.1) and cloned into the PUC57 plasmid, which was utilized as the standard containing the target nucleic acid fragment. All primers (Supplementary Table 2) used in the experiments were synthesized (Sangon Co., Ltd., Shanghai, China).

PCR was carried out using a Tiangen SuperReal Premix Plus Kit (Tiangen Biotech Co., Ltd., Beijing, China). The 50-μL PCR system comprised 25 μL of 2 × SuperReal Premix plus, 1.5 μL of forward and reverse primers (final concentration of 400 nM), 2 μL of template, and 20 μL of RNase-free ddH_2_O. The amplification was performed using a fluorescent quantitative PCR detection system (LineGene 9640, Hangzhou Bioer Technology Co., Ltd., Hangzhou, China) according to the following two-step procedure: 1 cycle at 95 °C for 15 min, followed by 40 cycles at 95 °C for 10 s, 60 °C for 20 s, and 72 °C for 32 s.

For PAGE, the gel was comprised of 6 mL 30% Acryl/Bis solution, 5.634 mL ddH_2_O, 240 μL 50 × TAE buffer, 6 μL TMEMD. Samples and 6 × ficoll gel loading buffer III were pre-mixed for 3 min, then added to the gel. Finally, standard PAGE (100 V, 110 min, 1 × TAE buffer) was performed with an EPS 300 electrophoresis apparatus (Tanon, Shanghai). Afterward, PAGE gel was stained in SYBR Green I (50 μL stock solution in 50 mL 1 × TAE buffer) for 30 min and imaged by 4100 digital gel image processing system (Tanon, Shanghai). All the reagents used for PAGE were provided by Sangon Corporation (Shanghai).

All the primers of oligonucleotides (Sangon Corporation provided) used in the study are analyzed by Primer premiere 5.0 and GeneRunner 6.5.51 software, listed in Supplementary Table 2. And the obtained PAGE results were analyzed by image J 1.51K software.

### Fabrication of the skin chip

The skin microfluidic chip was adopted from the model that we have established in our previous experiments^12^. The skin chip made of polydimethylsiloxane (PDMS, sylgard 184 silicone elastomer kit, Dow Corning Inc.) consists of three components: an endothedium-equivalence layer (bottom), a dermis-equivalence layer (middle), and an epidermis-equivalence layer (top). The upper, middle, and lower layers of the microfluidic chip were prepared by casting PDMS prepolymer on the master fabricated using photoresist (SU-8 2075, MicroChem). The silicon master was fabricated according to the conventional microfluidic photolithography technique, successively by casting, exposure, and developing. The detailed fabrication parameter was discussed in Supplementary Information (Supplementary Fig. 18 and Note 5).

CNE cells (Catalog No. BNCC341794) and HUVECS cells (Catalog No. BNCC337616) were purchased from Beina Chuanglian Biology Research Institute (Beijing, China). HACAT cells (Catalog No. CL-0090) and HFF cell (Catalog No. ZQ0450) were purchased from Procell Life Science & Technology Co., Ltd. (Wuhan, China) and Shanghai Zhong Qiao Xin Zhou Biotechnology Co., Ltd. (Shanghai, China), respectively. HeLa-GFP cells (HeLa Kyoto EGFP-H2B, Catalog No. 300673) were purchased from Cell Line Service (CLS, Germany). And all the cell lines were authenticated by cells supplier incorporations.

Then, three cell lines were cultured in the skin chip: HACAT cell on the top layer; HUVECS cell on the middle layer; HFF cell on the bottom layer. HACAT cell were cultured in MEM medium containing 15% FBS and 1% antibiotics (100 mg/mL streptomycin and 100 U/mL penicillin). HUVEC cell were cultured in CM-15 medium containing 89% F-12K, 0.1 mg/mL heparin, 0.05 mg/mL ECGs, 10% FBS, and 1% antibiotics (100 mg/mL streptomycin and 100 U/mL penicillin). HFF cell were cultured in Dulbecco’s modified Eagle’s medium with 10% FBS and 1% antibiotics (100 mg/mL streptomycin and 100 U/mL penicillin). All these cells were cultured in a cell incubator (37 °C, 5% CO_2_). The reagents for cell culture were purchased from Gibco (Thermo Fisher).

For cell 4′, 6-diamidino-2-phenylindole (DAPI) staining (Solarbio Corporation, Beijing, China), cells were washed twice with PBS for 1 min. DAPI solution (10 μg/mL) and cell complete growth medium (ratio of 1:10) was added for 40-min staining. Then, they were washed twice with PBS for 1 min. Finally, staining was observed on a fluorescence microscope (Ti-U, Nikon, USA) and a stereomicroscope (SMZ745T, Nikon, USA).

For HE staining of the skin chip (commercial kit provided by Sangon Biotechnology Corporation, Shanghai), it was used followed by the processes. First, three layers with different cells were washed by PBS for 1 time, and hematoxylin staining solution was added into each compartment for 5 min. All the layers were rinsed in running tap water for 1 min. Differentiate with 0.1% hydrochloric acid-ethanol for 10 s. Rinse in running tap water for 1 min. Bluing in PBST for 30 s. Rinse in running tap water for 1 min. Counterstain in eosin staining solution for 1 min. Rinse in running tap water for 1 min. Finally, observe cells under a stereomicroscope (SMZ745T, Nikon, USA).

### Setup of the wearable patch

The as-prepared three microneedles, including working, reference, counter, was attached to the anode side of the wearable iontophoretic patch via 3M tape. A flexible paper battery (Enfucell Flexible Electronics, Jiangsu, China) supplies power for the wearable iontophoretic patch. The integrated wearable system was available to laminated on human epidermis via 3M tape, with the penetration of microneedles (Supplementary Fig. 13). And gold wires of the three microneedles were connected to an electrochemical workstation, respectively. Finally, the real-time monitoring starts to record data. The application and usage of the wearables in real-world scenario was shown in Supplementary Fig. 13 and Movie 3.

### CNE Cell transfection and drug screening

We chose virus transfection in this study. Lentivirus package containing luciferase reporter and puro resistance gene was purchased from Jiman biotechnology co., LTD (Shanghai). According to protocol, briefly, 1 × 10^6^ TU/mL of lentivirus package in RPMI 1640 medium was mixed with 2 × 10^4^ of CNE cell in 24-well plate. And it was placed in the cell incubator (5% CO_2_, 37 °C) for 48 h. Afterward, the culture medium was removed. And fresh RPMI 1640 medium without lentivirus package was added and the cell was cultured for 48 h in the cell incubator (5% CO_2_, 37 °C).

For drug screening of transfected CNE cell, we chose puromycin (Yeasen Corporation, Shanghai) to screen those transfected CNE cells. To ensure the optimized concentration of puromycin, a series of puromycin concentration was set, such as 0, 3, 6, 9, 12, 15 μg/mL. The RPMI 1640 medium with different puromycin was added into each well. Finally, the optimized puromycin of 12 μg/mL was chosen through characterization.

### Animals experiments

Animals were cared for and maintained under the Guidelines of Laboratory Animals of Fudan University and approved by the Animal Ethics Committee of Fudan University, China (2021JSCHEM-020).

For every animal experiment, mice as animal models are chosen and the detailed information is listed as followed: the 4-week-old female Balb/c nude mice (*n* = 3 for each group) and 4-week-old KM mice (*n* = 3 for each group) are in the same housing condition, which is in the cycle of 6-h dark/18-h light (23 °C ± 2 °C, 30–40% of ambient humidity).

BALB/c nude female mice (aged 4 weeks) and KM female mice (aged 4 weeks) were purchased from Beijing Vital River Laboratory Animal Technology Co., Ltd. (Beijing, China). For EBV cfDNA in vivo monitoring, according to the reference, 500-μL volume of reprogramming CNE-Luc cells (2 × 10^6^ cells/mL, 250 μL) and BD matrigel (250 μL, Corning corporation) was subcutaneously injected into 8-week-old BALB/c nude mice^58,59^. The whole process was conducted at 4 °C.

For bioluminescence, isofluorane anesthesia was maintained using a nose cone delivery system during image acquisition. Mice bearing with CNE-Luc cells and negative control mice were injected with 100 µL of D-Luciferin potassium salt (30 mg/mL, Shanghai Yeasen Biotechnology Co., LTD). After 12 min, bioimaging signals were collected by CCD with exposure time of 60 s (in Vivo Xtreme, Bruker, USA).

For MN patch behavior upon target binding, the procedures were as followed. Before real-time test, anesthesia was applied to the mice (2% avertin, 250 μL for each subject). 2% avertin was prepared as followed: 0.625 g of 2,2,2-tribromoethanol (Sigma-Aldrich) was dissolved in 1.2 mL tert-amyl alcohol (Sinopharm Chemical Reagent Corporation, China), and 30 mL ddH_2_O was added. The solution was incubated in water-bath overnight (45 °C) and protected by foil. Mouse skin was cleaned by scrub cream and disinfected by 75% ethanol on a heating plate. Then, TE buffer was smeared on the region of interest close to anode side and dried by cotton. The conductive MNs was laminated on this region of interest. The work mode for animals consists of 3-min calibration, 3-min reverse iontophoresis, and 3-min biosensing signal collection.

Piglet skin was provided by the commercial breeder from piglets (domestic pigs, 1 month, Jingde Agricultural Products Co. Ltd). Piglet body skin was taken from the animals in slaughterhouse and kept at 4 °C during the transportation to our lab. After microneedle patch penetration, piglet samples were fixed in 4% paraformaldehyde fix solution (Sangon Corporation, Shanghai) overnight at 4 °C. Then the piglet samples were used for further characterization.

### Human trials

For human trials in Supplementary Fig. 15, participants were randomly recruited from research group members aged 19–37 years old (5 female participants and 5 male participants). This work was approved by Ethics Committee of Fudan University and complied with all relevant ethical regulations (IRB No. FE20037). Informed consent was obtained from all participants.

### Electrochemical measurements

Differential pulse voltammetry (DPV) testing was conducted. The scanning potential range was set from −0.2 V to 0.6 V at a scan rate of 50 mV/s in a 0.05 M K_3_[Fe(CN)_6_]/K_4_[Fe(CN)_6_] solution that contained 0.50 M KCl. The potential cut off for DPV was at 0.6 V.

Cyclic voltammetry (CV) measurement was conducted for electrochemical characterization. The scanning potential range was set from −0.2 V to 0.6 V at a scan rate of 50 mV/s in a 0.05 M K_3_[Fe(CN)_6_]/K_4_[Fe(CN)_6_] solution that contained 0.50 M KCl. Electrochemical impedance spectroscopy (EIS) was performed in the frequency range of 0.1 Hz to 100,000 Hz and at an alternating voltage of 5 mV, with an applied potential of 0.19 V, analyzed by Nova 1.7 and Z-view software. And *i*–*t* test was conducted with an initial potential of −0.1 V.

For the electrochemical measurements of the electrode, the commercial gold electrodes with relative modification membrane were used (inner diameter of 2 mm, outer diameter of 5 mm, working area of 3.14 mm^2^, length of 75 mm).

### AFM imaging

As for the AFM imaging of dCas9 on mica, the sample preparation was as followed. A fresh mica was washed by PBS for three times and sterilized by UV-light. 40 μL 0.5% APTES (Sigma-Aldrich) was added into the surface of mica for 3 min at room temperature. And the mica was washed by Milli-Q water and dried with nitrogen gas. 20 μL of 1 μM dCas9 was added into the center of the mica for 30 min at room temperature. Then, the mica was washed Milli-Q water and dried with nitrogen gas.

As for the AFM imaging of dCas9 on microneedles, the sample preparation was similar to that of CRISPR MNs in the section “Preparation of conductive microneedles”, without BSA blocking and sgRNA. Finally, the microneedles were cut into slice by a scalpel and attached on the iron wafer for AFM imaging.

The imaging of dCas9 on mica and microneedles in AFM was operated in PeakForce Quantitative Nanomechanical Mapping mode in air using super-sharp tips (SNL-10) at a scanning rate of 1.86 Hz.

### Statistics and reproducibility

Data are presented as mean values ± SD and analyzed by two-way ANOVA, calculated using Microsoft Excel 2016 version, and linear regression analyzed by using origin 2018 software. where *P* value significance is provided for figures. Significance level was implied by *, **, ***, ****, ns for *p* < 0.05, *p* < 0.01, *p* < 0.001, *p* < 0.0001, no significance, respectively. *N* values are also indicated within figure legends and refer to the independent test on the reproducibility of experiments. The animal sample sizes are chosen by randomization (three animals for each group), without blinding. The investigators were not blinded to allocation during experiments and outcome assessment.

### Reporting summary

Further information on research design is available in the Nature Research Reporting Summary linked to this article.

## Supplementary information

Supplementary Information

Peer Review File

Description of additional Supplementary File

Supplementary Movie 1

Supplementary Movie 2

Supplementary Movie 3

Reporting Summary

## Data Availability

The GenBank data used in this study are available from the NCBI database under accession codes M12294.2, NC001437.1, AF326573.1, A10072.1, and NC_000024.10 for WENV, JPEV, DENV, EBV, kidney transplantation, respectively. All the data generated and analyzed in the study are included in the paper and supplementary information. Source data are provided with this paper.

## Source data

Source Data
