# Supplementary material for: Programmable CRISPR-Cas9 microneedle patch for long-term capture and real-time monitoring of universal cell-free DNA
Source: Nat Commun. 2022 Jul 9;13:3999. doi: 10.1038/s41467-022-31740-3 (PMC9271037; doi:10.1038/s41467-022-31740-3)
Supplement: Supplementary file 1 — Supplementary Information [file 41467_2022_31740_MOESM1_ESM.pdf]

Supplementary Information

Programmable CRISPR-Cas9 Microneedle Patch for Long-Term Capture and Real-Time Monitoring of Universal Cell-Free DNA

Bin Yang, Jilie Kong*, Xueen Fang*

Department of Chemistry and Institutes of Biomedical Sciences, Fudan University, Shanghai, 200433, PR China

Corresponding author emails:

fxech@fudan.edu.cn (X. E. Fang)

jlkong@fudan.edu.cn (J. L. Kong)

Table of Contents

1. Supplementary Note 1. Theory for the wearable device signal output and the sensing mechanism
2. Supplementary Note 2. Biosafety of the wearable reverse iontophoretic patch
3. Supplementary Note 3. Biocompatibility and biosafety of the microneedles
4. Supplementary Note 4. Study on the selectivity of the CRISPR-based sensor
5. Supplementary Note 5. Fabrication and characterization of the skin microfluidic chip
6. Supplementary Note 6. The evaluation of dCas9 RNP made individually with sgRNAs for longitudinal monitoring of one target cfDNA
7. Supplementary Note 7. AFM observation of graphene nanoflakes on microneedles
8. Supplementary Note 8. Sensor to sensor variation
9. Supplementary Figure 1. Verification for the wearable device signal output
10. Supplementary Figure 2. Optimization and screening for sgRNA targeting to EBV cfDNA
11. Supplementary Figure 3. Construction for CRISPR microelectrode
12. Supplementary Figure 4. Validation for CRISPR-powered strategy
13. Supplementary Figure 5. Optimization for CRISPR reaction time on a microelectrode
14. Supplementary Figure 6. End-point CRISPR microelectrode for quantitative detection of EBV cfDNA
15. Supplementary Figure 7. EIS for CRISPR micro-electrode
16. Supplementary Figure 8. Construction and optimization for conductive microneedles
17. Supplementary Figure 9. SEM for graphene/chitosan membrane
18. Supplementary Figure 10. Mice bearing CNE-Luc cell under real-time monitoring
19. Supplementary Figure 11. Results of other three independent methods
20. Supplementary Figure 12. Slope of the plots of longitudinal target cfDNA in vitro
21. Supplementary Figure 13. The architecture, design, construction of the entire CRISPR-based microneedles biopatch
22. Supplementary Figure 14 The evaluation of the MNs swelling and dissolution on KM mice in vivo
23. Supplementary Figure 15. Biosafety of the wearable reverse iontophoretic patch
24. Supplementary Figure 16. Biocompatibility and biosafety of the microneedles

25. Supplementary Figure 17. Study on the selectivity of the CRISPR-based sensor
26. Supplementary Figure 18. Fabrication and characterization of the skin microfluidic chip
27. Supplementary Figure 19. The evaluation of dCas9 RNP made individually with sgRNAs for longitudinal monitoring of one target cfDNA
28. Supplementary Figure 20. Characterization of dCas9 on microneedles by AFM
29. Supplementary Figure 21. AFM observation of graphene nanoflakes on microneedles
30. Supplementary Figure 22. The comparison of conductivity for bare sensor, graphite sensor, and graphene sensor
31. Supplementary Figure 23. Page gel result of optimization for sgRNA targeting to sepsis and kidney transplantation cfDNA
32. Supplementary Figure 24. Stable sensitivity tests *in vitro*
33. Supplementary Figure 25. Sensor to sensor variation
34. Supplementary Figure 26. Stable sensitivity tests *in vivo*
35. Supplementary Table 1. CRISPR-Cas MNs compared with other MNs method in the field of chem-biosensing
36. Supplementary Table 2. Nucleic acid sequences in the experiments
37. Supplementary Reference

Supplementary Note 1. Theory for the wearable device signal output and the sensing mechanism

The sensing layer of the device was based on graphene. Graphene is a typical 2D material with Dirac cones, which particularly leads to ultrahigh carrier mobility[1]. And according to the reference[2], the electrolyte in the solution formed a Debye layer on the surface of the graphene biointerfaces, and it was a well-organized layer that could offset the difference in charge between the surface and the bulk solution. Then, Donnan potential occurred when the ion permeable layer separated two kinds of ions, as depicted in following Nernst formula equation 1,

$$\phi_D = \frac{RT}{F} In r_D \quad (1),$$

where r_D referred to Donnan equilibrium constant.

For an as-prepared CRISPR electrode, there were two phases in the system: CRISPR containing dRNP and target DNA on graphene, epidermis microenvironment containing electrolytes. The immobilized compound layer of graphene, proteins, sgRNA, and nucleic acids separated the graphene channel and double-layer from the epidermis microenvironment. The charge that generated a net charge in an immobilized ion-permeable layer required additional accumulation of counter ions to maintain charge neutrality. Thus, the difference between the epidermis microenvironment and the immobilized ion permeation layer produced the Donnan potential, as depicted in following equation 2,

$$\phi_D = \frac{RT}{F} In\left[\frac{\sqrt{4C_S^2 + C_{CRISPR}^2} + C_{CRISPR}}{2C_S}\right] \quad (2),$$

where C_s, C_{CRISPR} referred to the epidermis microenvironment and immobilized layers, respectively.

Supplementary Note 2. Biosafety of the wearable reverse iontophoretic patch

The skin irritation caused by reverse iontophoresis is one of the biosafety for medical device. Early in 2019[6], it was reported that reverse iontophoresis had no evident impact on human blood pressure and pulse. Then, it had also been reported that the wearable reverse iontophoretic patch modified with conductive silver ink could be used to sample sweat and ISF biomarkers[7]. Hereby in this study, we utilized multi-walled carbon nanotubes conductive ink for patterning the circuit of reverse iontophoresis. To access this, we conducted the printed wearable reverse iontophoretic patch on piglet skin in different period and collected piglet skin sample for histological analysis. As shown in **Supplementary Figure 15a**, compared with the NTC group (0 min), the histological analysis of the local tissue revealed that there is hardly obvious tissue damage with wearable reverse iontophoretic patch during 80 min administration. Equally, the collected piglet skin was stained with terminal deoxynucleotidyl transferase-mediated dUTP nick-end labeling (TUNEL) and 4',6-diamidino-2-phenylindole (DAPI). The number of apoptotic cells in the testing group (10 min, 20 min, 40 min, 80 min) was comparable to that of the control group (0 min), indicating that the as-prepared wearable reverse iontophoretic patch (10 V) had little influence on skin.

In this study, to vividly reflect upon the skin irritation on human epidermis, the assessment of pain or discomfort perception was implemented via visual analog scale (VAS, score range from 0 to 10). Ten volunteers were blinded to the presence of reverse iontophoresis process (10 V, 10 min) during the experiments, as it might hindered the discerning pain perception and yield higher scores through VAS. In **Supplementary Figure 15b** and **15c**, the average VAS scores were reported: 0.1±0.3 of wearable patch without reverse iontophoresis; 0.6±0.6 of wearable patch with reverse iontophoresis. From the results of visual photographs (**Supplementary Figure 15b**), there is little skin irritation occurred on 10 participants. Even though the participants of the study were not informed of reverse iontophoresis in the patches for all applications, one participant gave a higher VAS score (2 score). This is probably due to individual difference caused by permeability and conformality of the PDMS film. Collectively, the above results indicated the little skin irritation on epidermis during the short-term reverse iontophoresis administration.

Supplementary Note 3. Biocompatibility and biosafety of the microneedles

We then sought to test biocompatibility and biosafety in vivo of the microneedles. In this study, KM mouse (4-week-old, female) was chosen as model, on account that it had immune system which could reflect upon the original biocompatibility of the graphene microneedles. From the visual results of **Supplementary Figure 16a**, it can be seen that there is little skin immune response on three KM mice. Then, histological examination of the local tissues (the graphene MNs application site) collected from KM mice revealed that the tissue damage of the tested groups was consistent with NTC group (**Supplementary Figure 16b**).

Meanwhile, the biosafety of the microneedles was verified via cytotoxicity tests, including PMVE/MA, graphene/chitosan@PMVE/MA. Cytotoxicity tests were assessed by 3-(4,5-dimethylthiazol-2-yl)-2,5-diphenyltetrazolium bromide (MTT) assays after incubation with HeLa-GFP cells for 28 h. As shown in **Supplementary Figure 16c and 16d**, these two materials had little significant cytotoxic effect on the viability and morphology of HeLa-GFP cells after 28 h of treatment. Even though the concentration was over 5000 μg/mL (equivalent to 112.6 PMVE/MA needles, 80 graphene/chitosan@PMVE/MA needles, respectively), cell viability was more than 85 %, indicating good biosafety of the microneedles.

Supplementary Note 4. Study on the selectivity of the CRISPR-based sensor

The specific recognition ability in this study to exclude the interference is one of the most significant bioanalytical parameters, affected by the complexity of the matrix. To evaluate the selectivity of the assay, according to the reference, we chose a range of common interferences existed in ISF for the CRISPR-based sensor operation (**Supplementary Figure 17**). Compared to the current response of 0.35 nM EBV cfDNA target, none of these interferences displays an obvious response (2 mM), with an RSD% of 2.2%, 4.1%, 2.3%, 2.4%, 2.3%, 2.4% for Na^+, K^+, Cl^-, glucose, urea, BSA, respectively. Even in a higher concentration of glucose (30 mM, over the level of diabetic patient) and NaCl (154 mM, the equal concentration of saline), it showed significant differences (P value of 0.000193 for glucose, P value of 0.000792 for NaCl), compared with positive group. It proved the good selectivity of the assay for recognizing the target cfDNA.

Supplementary Note 5. Fabrication and characterization of the skin microfluidic chip

In this section, the skin-mimic microfluidic chip was adopted from the model that we have ever established in our experiment. As shown in **Supplementary Figure 18a**, the skin chip made of polydimethylsiloxane (PDMS, sylgard 184 silicone elastomer kit, Dow Corning Inc.) consists of three components: an endothedium-equivalence layer (bottom), a dermis-equivalence layer (middle), and an epidermis-equivalence layer (top). The upper, middle and lower layers of the microfluidic chip were prepared by casting PDMS prepolymer on the master fabricated using photoresist (SU-8 2075, MicroChem). The silicon master was fabricated according to the conventional microfluidic photolithography technique, successively by casting, exposure, developing.

The size of the upper and bottom layer was designed to allow HACAT and HFF cells to be cultured (60 mm×60 mm×240 μm, length×width×height). And the middle layer was mimicking dermis consisting capillaries and vessels for HUVECS cells attachment and collagen, thus the channel size was 50 mm×500 μm×240 μm, length×width×height). A PDMS precursor mixture at a weight ratio of base to curing agent of 10:1 was poured carefully onto the master after surface hydrophobic treatment, which was stirred for 15 min and vacuumed for 15 min to eliminate bubbles and cured at 80 °C for 4 h. Then, the cured PDMS replica was gently peeled off from the silicon master. Hole punchers and scalpels were manually used to drill the shaped inlets, outlets, and gas vents. Porous membranes were placed between each layer to separate the chambers by plasma-treated method. The porous membranes were sealing membrane paper. Three parts are bonded together by plasma treatment and connected to the flow syringe pump as well as a CO_2 generation incubator to form a complete bio-microfluidic system. The device was inspected under a microscope to reconfirm inlets/outlets, microchannels and gas vents. Before cell culture, the skin chip was sterilized with 75 % ethanol and washed with PBS three times. The bottom was pipetted with a HFF suspension (10^5 cells/mL) and five microliters of 10 % collagen (type I, PBS buffer). The middle layer was pipetted with a HUVECS suspension (10^5 cells/mL) and five microliters of 10 % collagen (type I, PBS buffer) for each channel. The upper layer was pipetted with a HACAT suspension (10^5 cells/mL). Afterwards, the chip was incubated at cell culture incubator (37 °C, 5% CO_2) for 24 h. Before the simulation, DMEM medium in the upper layer was removed and this layer was washed with PBS. Then, the simulated ISF solution containing target DNA was added into the layer via a

mini flow springe-pump. Therefore, it could be used for a series of simulated experiments with a flow rate of 7.2 μL/min by a mini flow syringe pump.

According to the results of **Supplementary Figure 18b and 18c**, compared with NTC group, the cell morphology and integrity were well-defined in the testing group, indicating that the established skin chip could be utilized for the following in vitro applications and iontophoresis has hardly impact on skin.

Supplementary Note 6. The evaluation of dCas9 RNP made individually with sgRNAs for longitudinal monitoring of one target cfDNA

Although we focused on the detection of EBV cfDNA at the genomic site of sgRNA5 in the study, the established method could be applicable to other genomic sites. To end this, we identified four sgRNA to fabricated CRISPR-Cas9 sensor for monitoring target DNA, including sgRNA1 (reverse), sgRNA2 (forward), sgRNA3 (reverse), sgRNA5 (forward). In **Supplementary Figure 19**, compared with the positive group (0.35 nM EBV cfDNA), each sgRNA groups showed little current response to the interferences (2 mM), with significant differences, indicating that the proposed CRISPR-based sensor had well-defined selectivity. These results primarily deduced that the proposed detection method in this study might be used for a longitudinal monitoring of one target cfDNA.

Supplementary Note 7. AFM observation of graphene nanoflakes on microneedles

To vividly observe the graphene nanoflakes structure formed atop microneedles, AFM imaging was conducted for mica and microneedles simultaneously. From the results (**Supplementary Figure 21**), compared with conventional sample preparation on the mica, it was observed that some wrinkles were existed on the surface of the microneedles. From the section analysis, we found that the height of graphene nanoflakes on the microneedles was ~14 nm, while that on mica was ~7 nm, probably due to the tilt angle for microneedles surface. After incubation in simulated ISF solution (PBS, 37 °C, 30 min), the height of graphene nanoflakes wrinkles increased on mica and microneedles. To further ensure the graphene nanoflakes structure, graphite on mica was also observed via AFM imaging. It was found that it consisted of continuous monolayer graphene nanoflakes, with a height of ~245 nm. These results deduced that the graphene nanoflakes/chitosan was successfully modified on the microneedle surface via drop-casting method.

Supplementary Note 8. Sensor to sensor variation

In order to evaluate the variation of the response of sensor-to-sensor in the presence of 3.59 nM target EBV cfDNA, five different CRISPR microneedles sensor were prepared in different batches. As shown in **Supplementary Figure 25**, there was no significance (P value=0.33) among five different sensors, with the coefficient of variation (C.V.) of 9.34%, indicating a well-defined sensor-to-sensor variation.

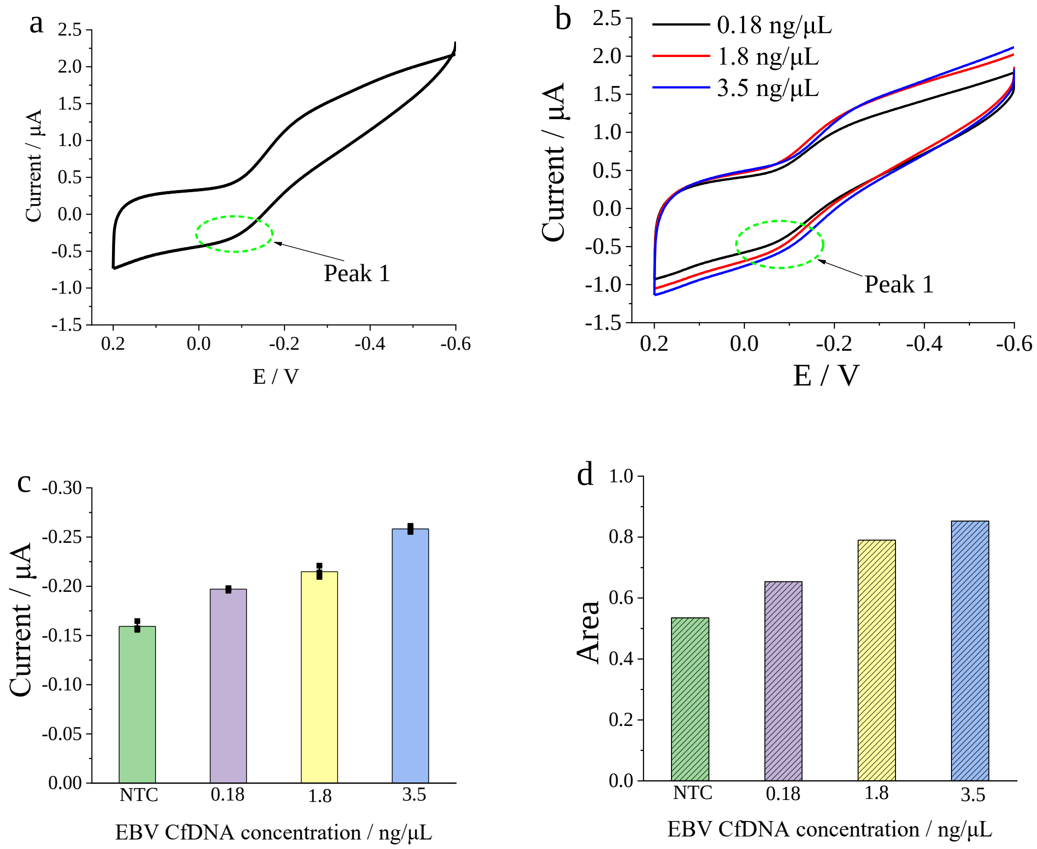

Supplementary Figure 1. Verification for the wearable device signal output. (a) CV plot for the as-prepared CRISPR micro-electrode without EBV cfDNA, scanning rate of 0.05 V/s, sampling interval of 0.001 V. (b) CV plots for CRISPR micro-electrode under different concentration of EBV cfDNA, scanning rate of 0.05 V/s, sampling interval of 0.001 V. (c) Peak 1 current of CV plots under different conditions, data presented as mean values ± SD, n=3 replicated measurements. (d) Integration area of CV plots under different conditions. From the data, when the as-prepared CRISPR micro-electrode was dipped in to PBS buffer (0.01 M, pH 7.4), a characteristic peak 1 around -0.1 V was observed. Due to Donnan potential, the peak 1 current increased, as the concentration of EBV cfDNA increased. Additionally, the integration area increased, as the concentration of EBV cfDNA increased, indicating that the immobilized layer had a well-defined capacitance and electrochemical performance. All the data was analyzed by Origin software. Thus, we chose the initial potential of -0.1 V for the subsequence real-time monitoring experiments.

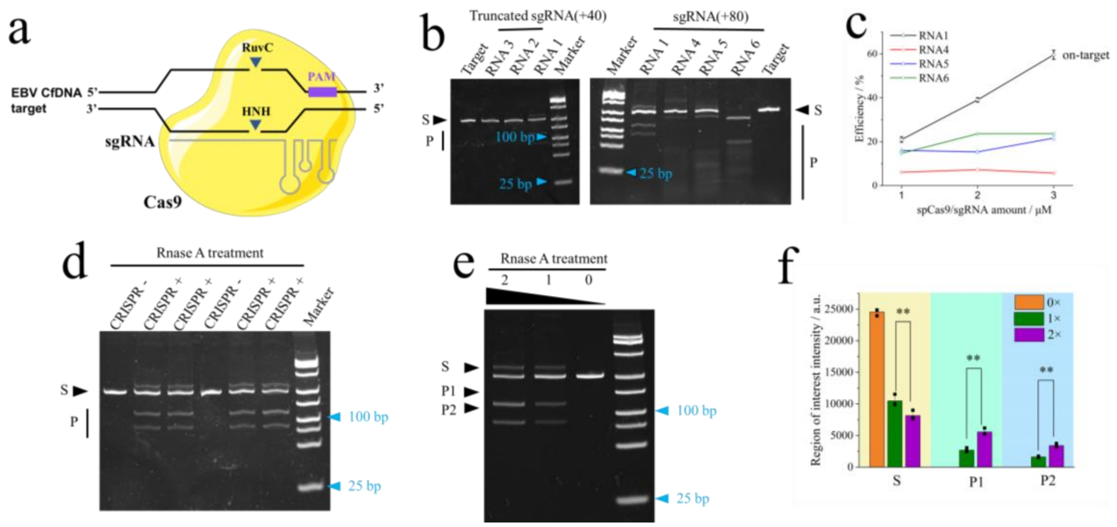

Supplementary Figure 2. Optimization and screening for sgRNA targeting to EBV cfDNA. (a) Schematic for CRISPR-Cas9 gene editing to EBV cfDNA. (b) Comparison of two types of sgRNA. According to reference[3,4], truncated sgRNA (+40) has a relative low efficiency for gene editing. It was validated by PAGE gel results in this study. We found that a complete sgRNA show high efficiency for EBV cfDNA gene editing. (c) Efficiency for four complete sgRNA, data presented as mean values ± SD, n=3 independent experiments. (d) Reproducibility of the CRISPR-Cas9 targeting to EBV cfDNA. (e) PAGE gel of sgRNA/Cas9 targeting to EBV cfDNA by variable amount, 0, 1, 2 referring to 0×, 1×, 2×sgRNA/Cas. (f) Region of interest intensity from the PAGE gel of sgRNA/Cas9 targeting to EBV cfDNA by variable amount, 0, 1, 2 referring to 0×, 1×, 2×sgRNA/Cas9 respectively, *p<0.05, **p<0.01, ***p<0.001, ****p<0.0001, analyzed by two-way ANOVA, p value of 0.0024, 0.0014, 0.0041 for S, P1, P2 respectively, data presented as mean values ± SD, n=3 independent experiments.

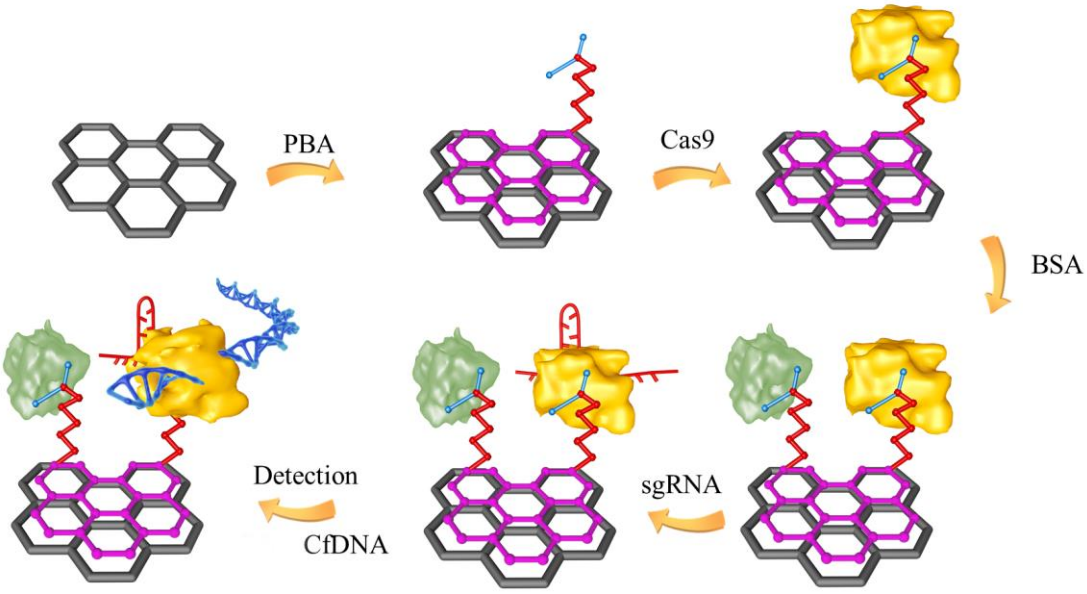

Supplementary Figure 3. Construction for CRISPR microelectrode. The method was similar to CRISPR microneedles construction other than 1-pyrenebutanoic acid (PBA). PBA was stacked on graphene via π-π aromatic stacking.

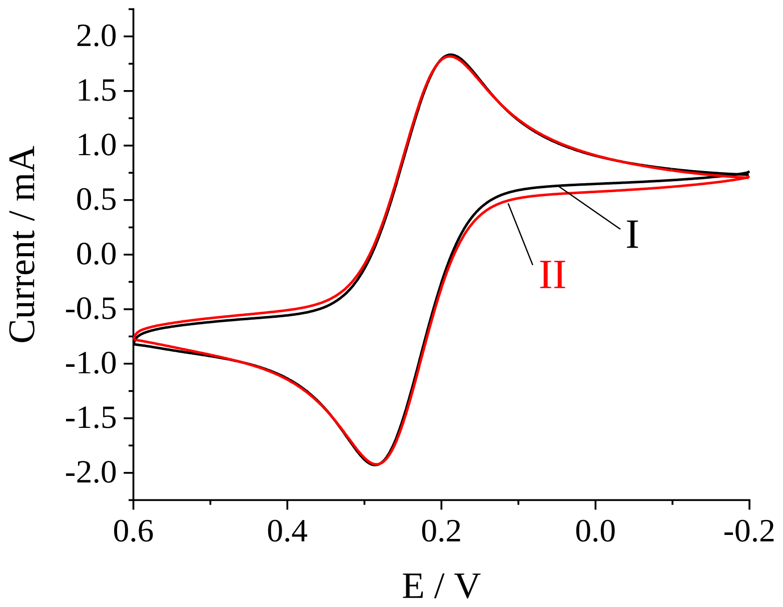

Supplementary Figure 4. Validation for CRISPR-powered strategy. Curve I and II referring to micro-electrode after modified graphene and PBA, graphene and PBA modified micro-electrode targeting to 2×10^{-11} M EBV cfDNA, respectively. In order to test if the non-specific adsorption and physical adsorption of DNA exerts impact on the signal output, PBA was modified on the graphene electrode, and incubated in a certain concentration of EBV cfDNA solution at 37°C for 20 min. The interface was washed with distilled water for 5 minutes, and finally CV was scanned using 0.05 M $[Fe(CN)_6]^{3-/4-}$. As shown, the two curves had hardly obvious change in current and peak deviation. This further showed that EBV cfDNA was recognized and captured by the interface through the CRISPR-driven reaction for gene editing.

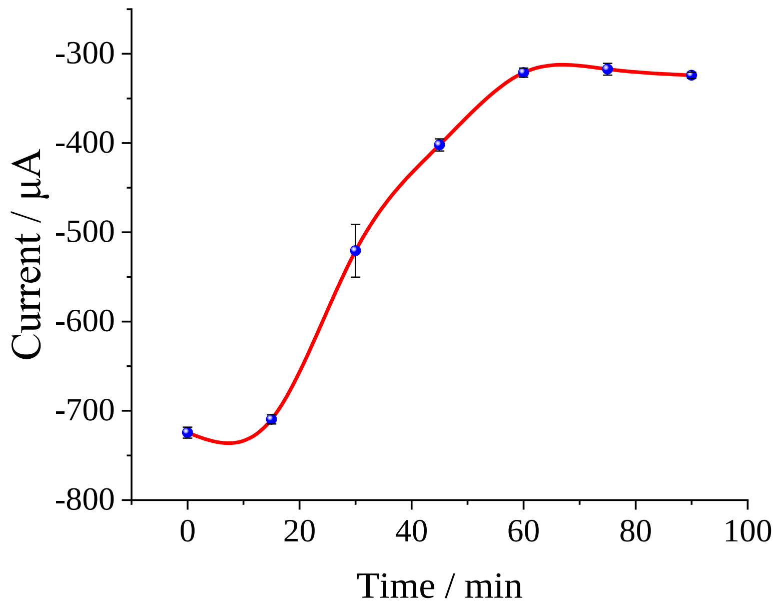

Supplementary Figure 5. Optimization for CRISPR reaction time on a microelectrode. Under the above preliminary conditions, the reaction time of CRISPR-Cas9 was optimized. EBV cfDNA as the target to explore the changes in the electrode interface, using 0.05 M $[Fe(CN)_6]^{3-/4-}$ electrochemical probe, data presented as mean values ± SD, n=3 independent experiments. As shown, it is found that as the CRISPR reaction proceeded, the resistance of the electrode surface continues to increase, and the current value continued to decrease. This may be due to the fact that the spatial conformation of the dCas9 protein changed with the CRISPR reaction, affecting the electron transfer and conductivity of the electrode interface. It can be seen that the CRISPR reaction became faster from 30 minutes, and after 60 minutes, the current value remained basically stable, so we chose 60 minutes as the optimized end-point reaction time for micro-electrode.

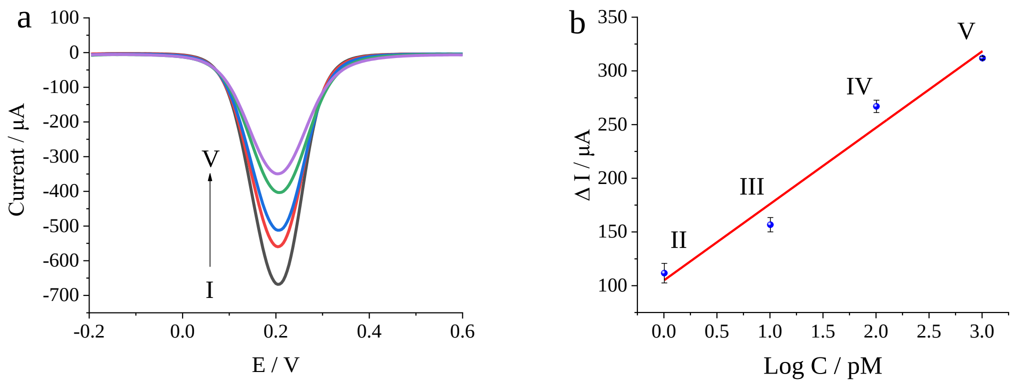

Supplementary Figure 6. End-point CRISPR microelectrode for quantitative detection of EBV cfDNA. (a) Dynamic curves for different concentration of EBV cfDNA, I to V referring to 0, 1×10^{-9} M, 1×10^{-10} M, 1×10^{-11} M, 1×10^{-12} M. (b) Calibration curve, data presented as mean values ± SD, n=3 independent experiments. A linear relationship was observed between the change in current and EBV cfDNA concentration in the range of 1 nM-1 pM with the equation: ΔI (μA)=0.7107·lgC+1.0488 (R=0.9836), with a detection limit of 1 pM (DL = $3\delta_b/K$).

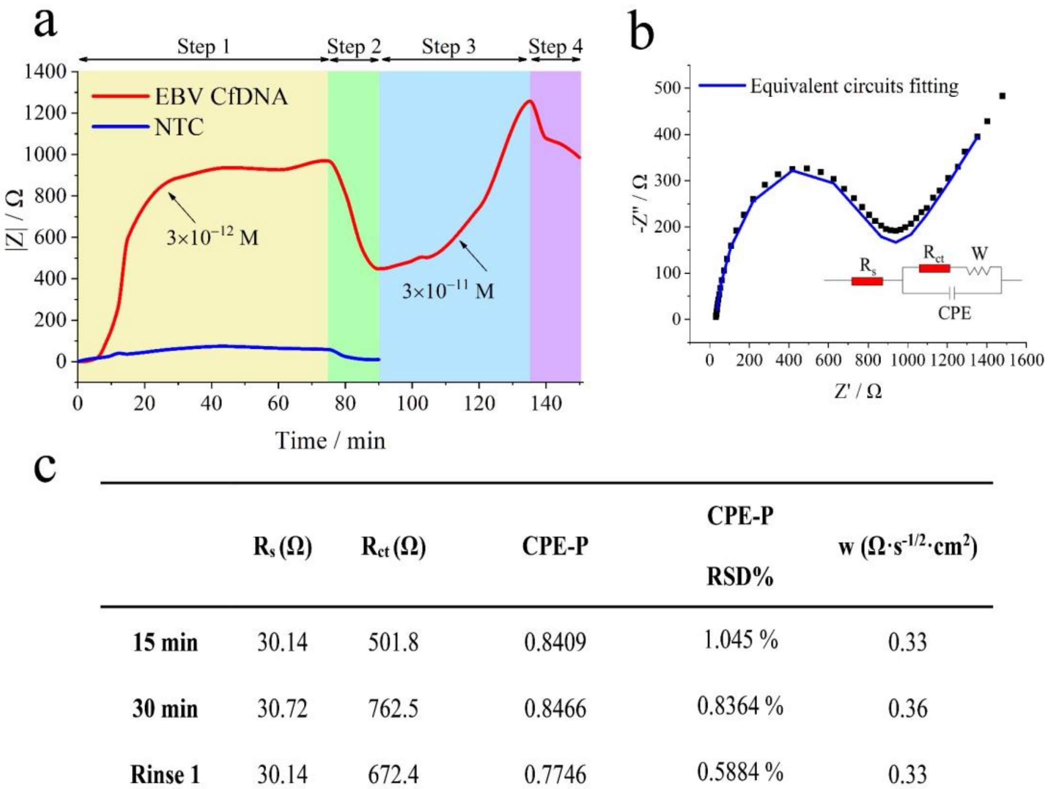

c

	R_s (Ω)	R_{ct} (Ω)	CPE-P	CPE-P RSD%	w ($\Omega \cdot s^{-1/2} \cdot cm^2$)
**15 min**	30.14	501.8	0.8409	1.045 %	0.33
**30 min**	30.72	762.5	0.8466	0.8364 %	0.36
**Rinse 1**	30.14	672.4	0.7746	0.5884 %	0.33

Supplementary Figure 7. EIS for CRISPR micro-electrode. (a) Real-time EIS curve under reverse iontophoresis, using 0.05 M $[Fe(CN)_6]^{3-/4-}$ electrochemical probe, step 1-4 referring to monitoring of 3×10^{-12} M EBV cfDNA, rinse 1 by TE buffer (37 °C，pH 8.0), monitoring of 3×10^{-11} M EBV cfDNA, rinse 2 by TE buffer (37 °C，pH 8.0). (b) Simulation of EIS curve for 30-min time point. (c) Simulation results of the CRISPR micro-electrode capacitance performance. The 30-min group had a CPE value of 0.8466 (RSD=0.8364%). Three groups at different time point showed a CPE value over 0.5. These results evidently showed that the CRISPR sensitive membrane on the micro-electrode had a well-defined capacitance.

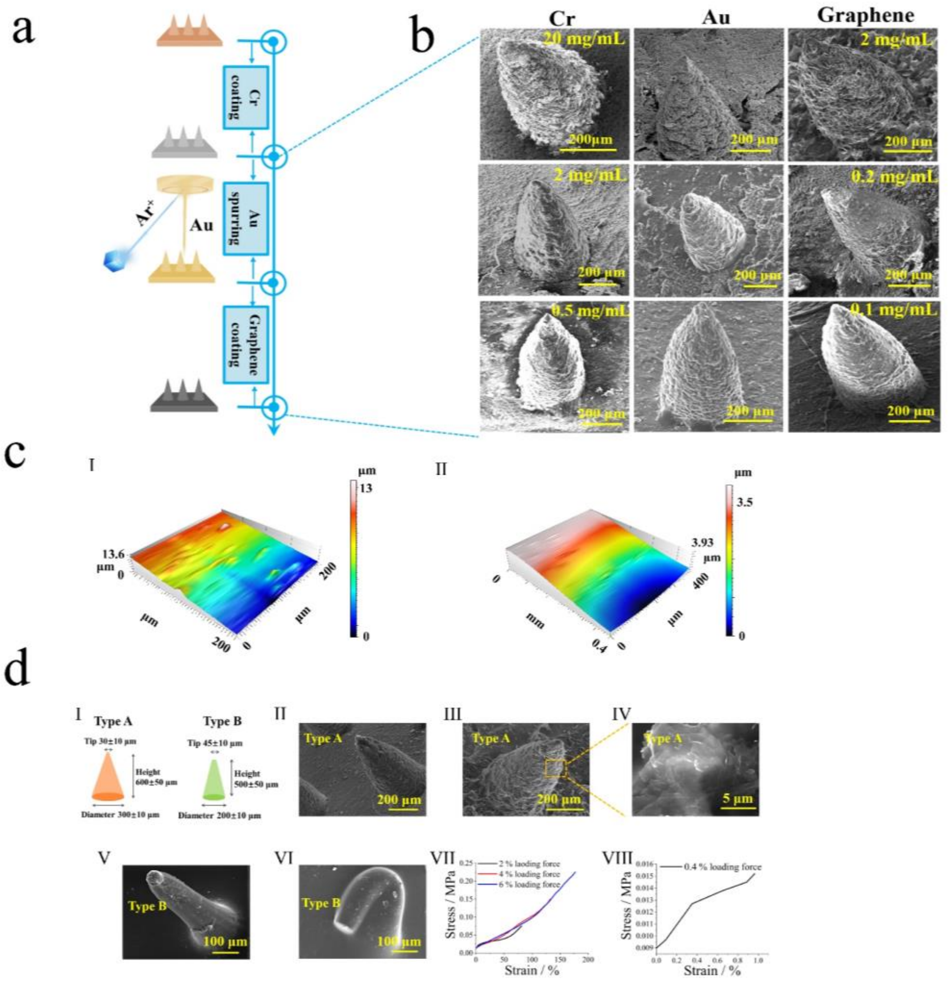

Supplementary Figure 8. Construction and optimization for conductive microneedles. (a) Schematic of the conductive microneedles process. (b) SEM result of the microneedles modification. The optimized concentration of Cr and graphene was 0.5 mg/mL and 0.2 mg/mL respectively. According to the equation d=KIVT, a theoretical thickness of Au spurring was 102 nm under the optimized condition (600 s, 10 mA, argon), the results obtained from three independent repeated experiments. (c) Stylus profiler for Cr+Au (I) and Au (II) functionalized membrane, 0.05 mg loading force, 0.1 mm/s scanning speed. (d) Comparison of different shape microneedles in mechanical performance. I to VIII referring to schematic of two types, type A before compression test, type A after compression test (60 N), type B before compression test, type B after compression test (6 N), different loading (20 N, 40 N, 60 N) for type A, 4 N loading for type B, the results obtained from three independent repeated experiments. These results showed that shape, height, and base diameter had of microneedles had impact on its rigidity, stiffness, and penetration ability.

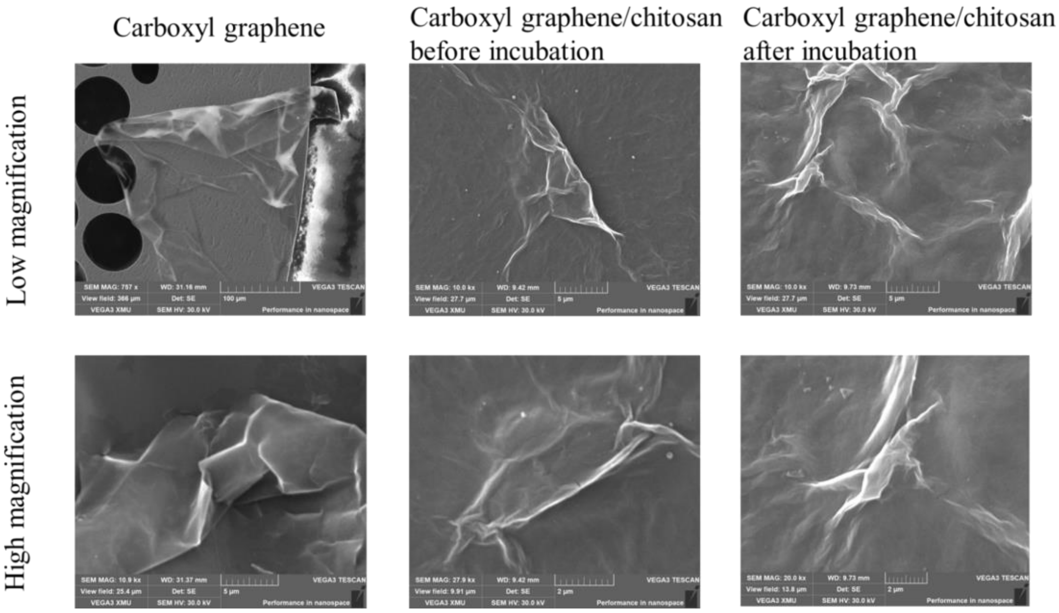

Supplementary Figure 9. SEM for graphene/chitosan membrane. The results obtained from three independent repeated experiments.

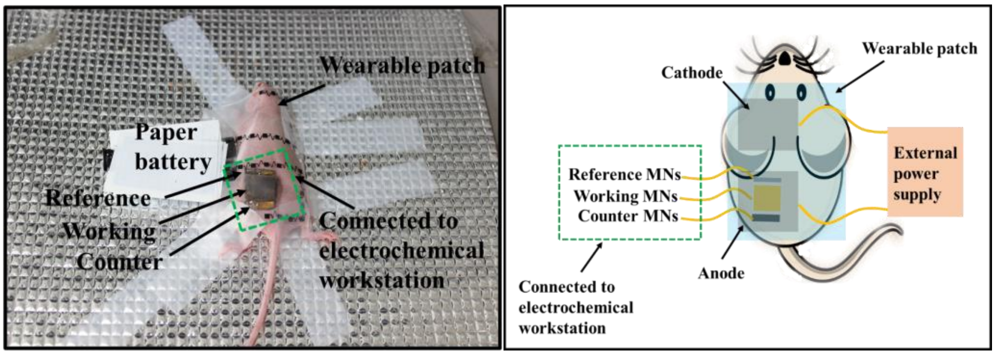

Supplementary Figure 10. Mice bearing CNE-Luc cell under real-time monitoring. left: optical photograph, right: schematic photograph.

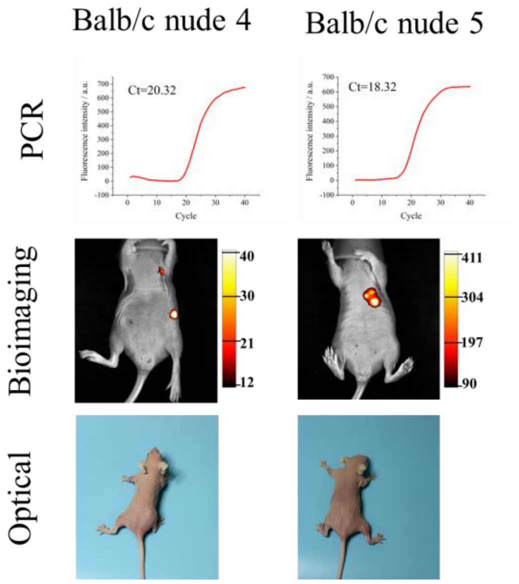

Supplementary Figure 11. Results of other three independent methods.

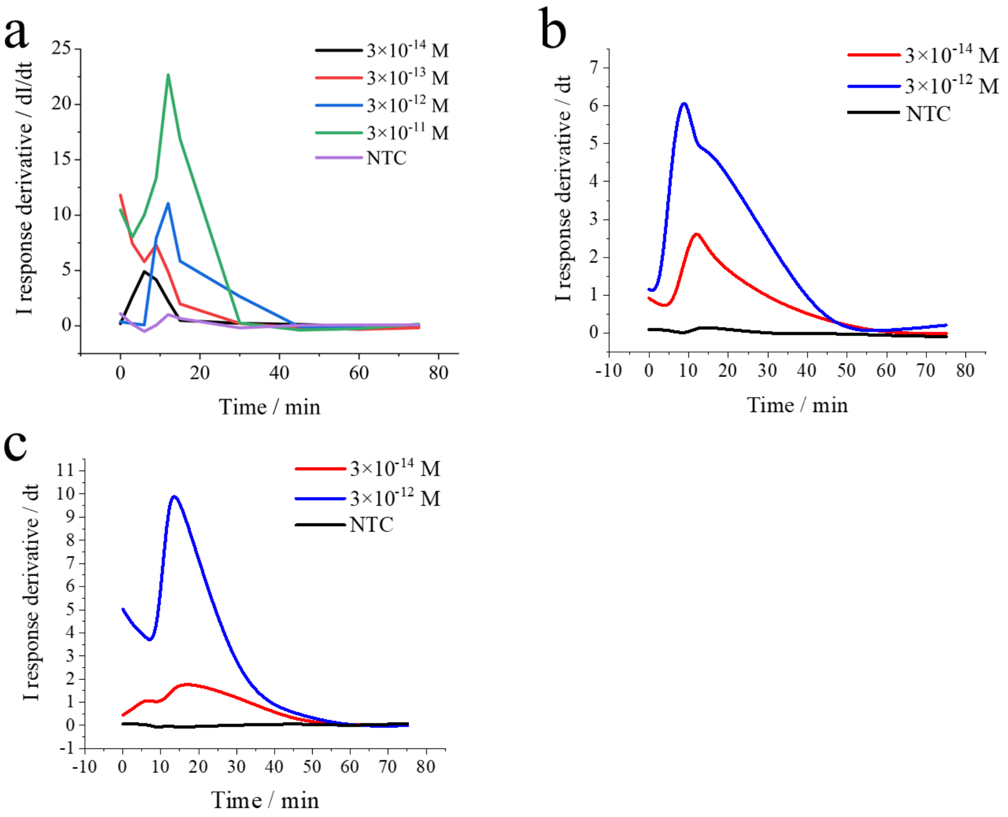

Supplementary Figure 12. Slope of the plots of longitudinal target cfDNA in vitro. Calculated by simple differentiation, including (a) EBV cfDNA, (b) sepsis cfDNA, and (c) kidney transplantation cfDNA.

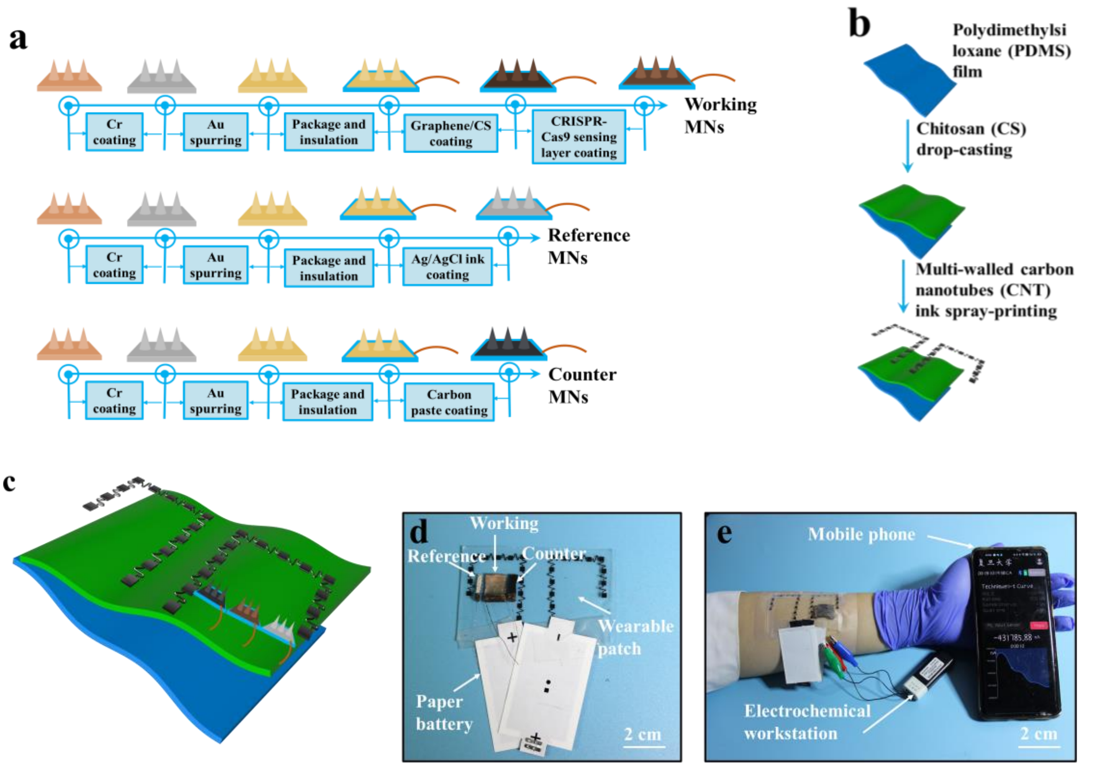

Supplementary Figure 13. The architecture, design, construction of the entire CRISPR-based microneedles biopatch. (a) The workflow of three-electrode system MNs patch. After package and insulation, the MNs were attached a gold wire (diameter of 200μm) that was connected to electrochemical workstation. (b) The workflow for iontophoretic wearable patch construction. 1% chitosan solution was drop-casted on the surface of the PDMS film to enhance hydrophilicity, then multi-walled carbon nanotubes ink was spray-printed on the surface which was used for reverse iontophoresis. (c) The integration of three MN patch and wearable iontophoretic patch. (d) The visual photograph of the integrated wearable system with external power supply (paper battery, Enfucell Flexible Electronics, Jiangsu, China). (e) The integrated wearable system was connected to a portable electrochemical workstation (Refresh Inc., Shenzhen, China) and signal was wirelessly transmitted to a mobile phone via Bluetooth. The integrated wearable biopatch was attached on a healthy volunteer and i-t raw data was displayed on mobile phone.

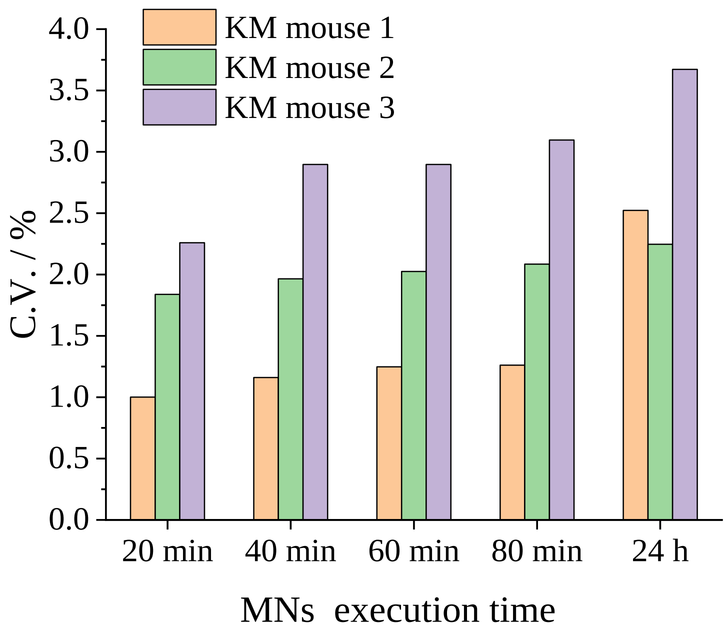

Supplementary Figure 14 The evaluation of the MNs swelling and dissolution on KM mice in vivo. The C.V. of the three MNs tested on KM mice in vivo, n=3 biologically independent animals. The PMVE/MA/PEG bare MNs was modified with Cr film, gold film, successively. Then it was drop-casting graphene/chitosan solution. Finally, the as-prepared graphene MNs was laminated on healthy KM mice (four-week-old, n=3 biologically independent animals). From the results, it showed that there is hardly obvious change of water absorption for three graphene MNs (maximum C.V.< 3.6%) in 24h, which implied the feasibility for the sensing capability, because the execution time of the CRISPR-based MNs was within 75 min.

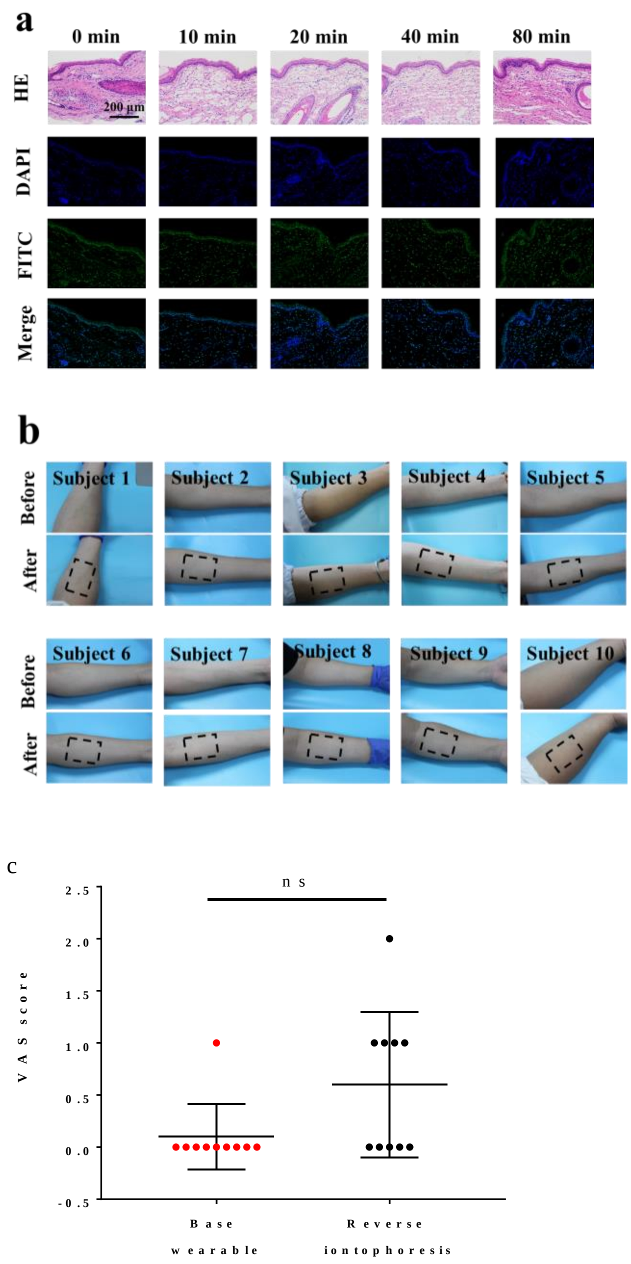

Supplementary Figure 15. Biosafety of the wearable reverse iontophoretic patch. (a) Histological analysis of piglet skin after reverse iontophoresis at different time point, stained with HE and TUNEL, the results obtained from three independent repeated experiments. (b) Visual photograph of the volunteered participants attached with wearable reverse iontophoretic patch (10 V, 10 min). (c) VAS score reported by the volunteered participants for base wearable (without reverse iontophoresis) and reverse iontophoresis (the printed wearable patch), data presented as mean values ± SD, n=10 biologically independent samples.

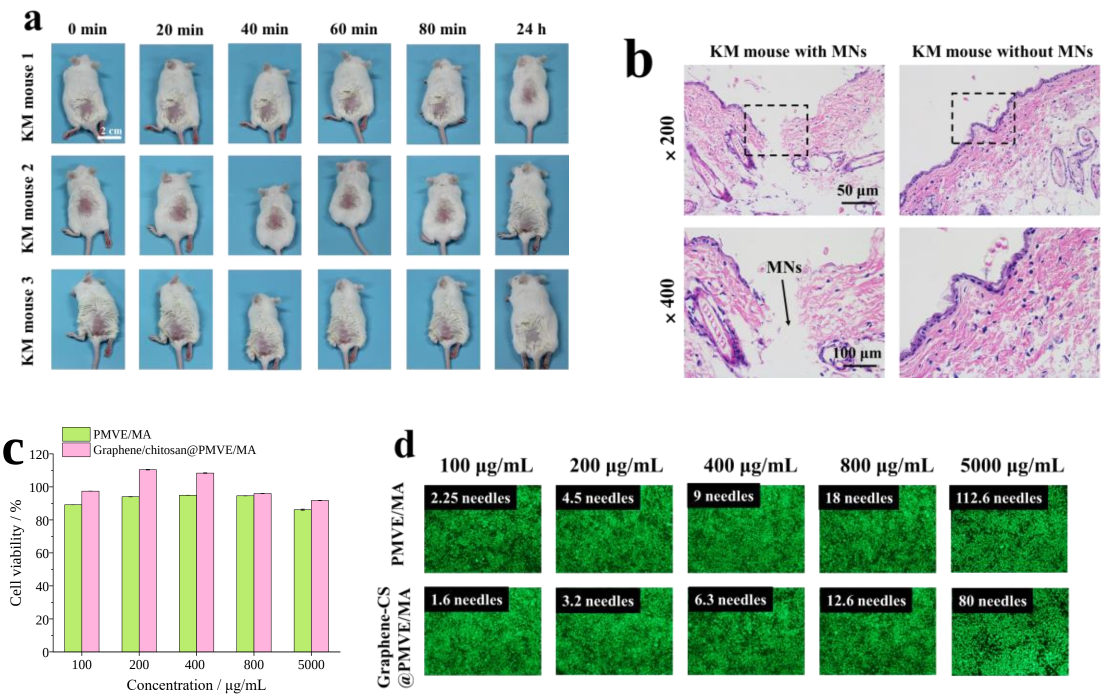

Supplementary Figure 16. Biocompatibility and biosafety of the microneedles. (a) Photograph of the KM mice after MNs execution in different time point. (b) Histological analysis of KM mouse after graphene MNs administration, stained with HE. (c) Viability of HeLa-GFP cells after treatment with the PMVE/MA and graphene/chitosan@PMVE/MA, respectively for 28 h, according to an MTT assay with PBS as a blank (n=5 independent experiments). (d) Fluorescence imaging photograph of MTT assays with PMVE/MA and graphene/chitosan@PMVE/MA, respectively, the concentration unit was converted to the quantity of microneedles, n=5 independent experiments.

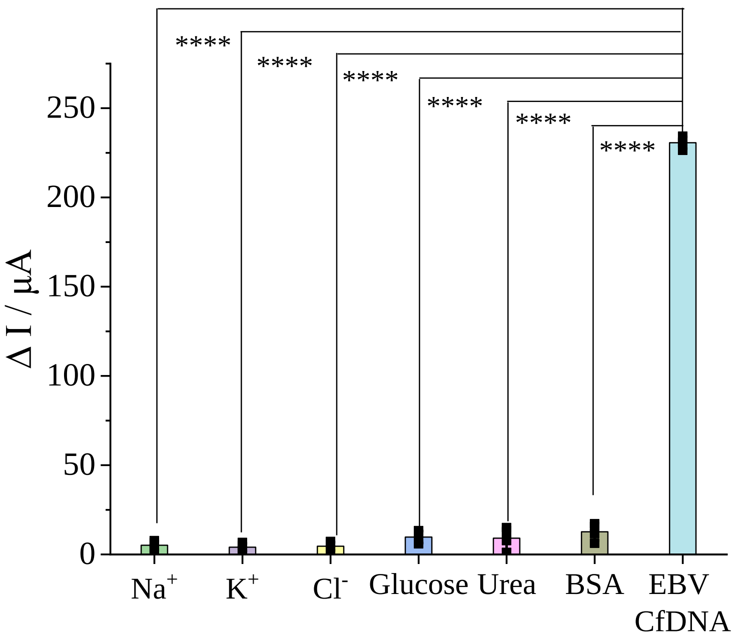

Supplementary Figure 17. Study on the selectivity of the CRISPR-based sensor. The concentration of the interferences was 2 mM, while that of the EBV cfDNA target was 0.35 nM, *p<0.05, **p<0.01, ***p<0.001, ****p<0.0001, analyzed by two-way ANOVA, p value of 0.0000032, 0.0000025, 0.0000028, 0.0000058, 0.000017, 0.000012 for $Na^{+,}$ K^{+}, Cl^{-}, glucose, urea, BSA respectively, data presented as mean values ± SD, n=4 independent experiments.

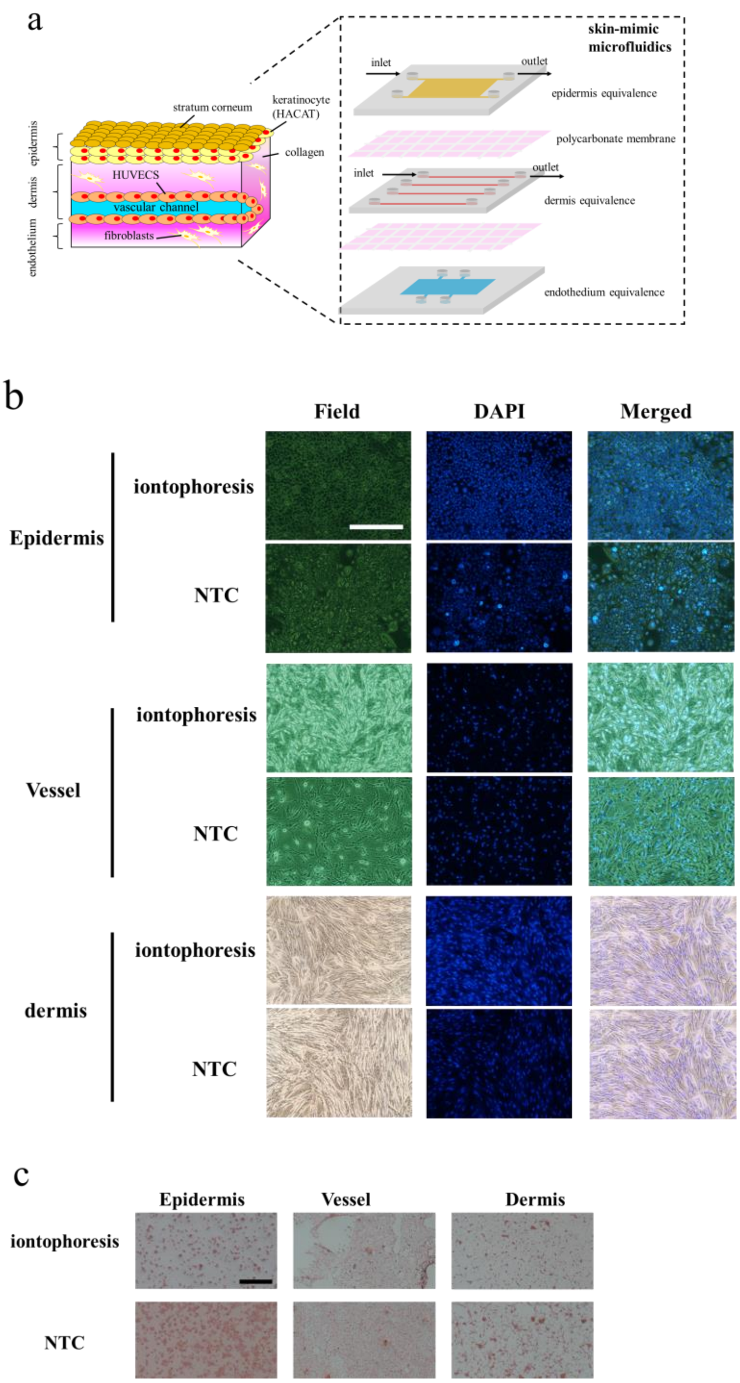

Supplementary Figure 18. Fabrication and characterization of the skin microfluidic chip. (a) Schematic of the skin-mimic microfluidic chip *in vitro* experiments. (b) Fluorescence microscope images of the skin chip, blue referring to DAPI staining for HACAT, HUVECS, HFF cells, under iontophoresis process (15 min, 10V), the results obtained from three independent repeated experiments, scale bar=100 µm. (c) HE staining for the skin chip, under iontophoresis process (15 min, 10V), the results obtained from three independent repeated experiments, scale bar=500 µm.

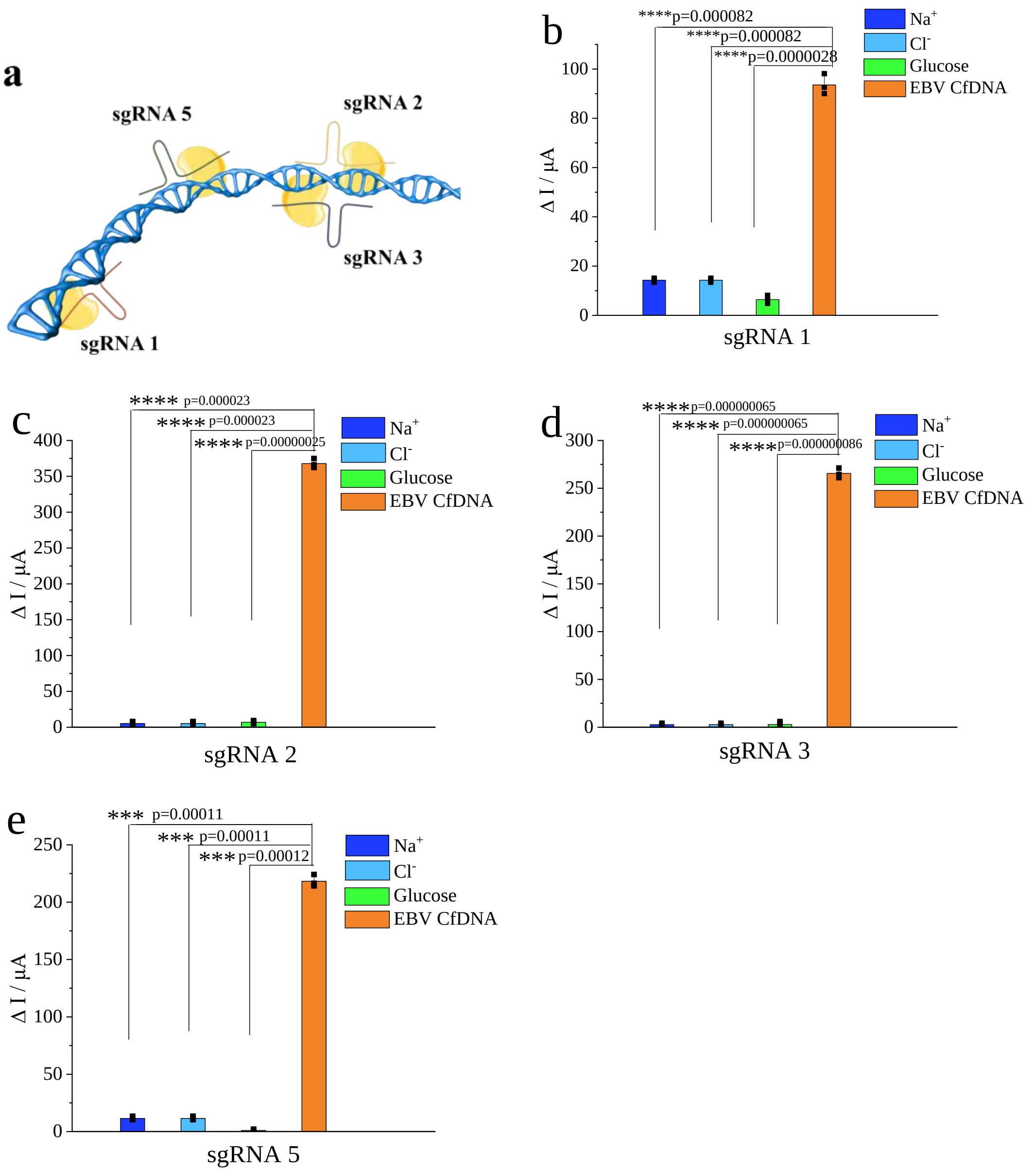

Supplementary Figure 19. The evaluation of dCas9 RNP made individually with sgRNAs for longitudinal monitoring of one target cfDNA. (a) Schematic of a dCas9-sgRNA complex binding target cfDNA via sgRNA1, sgRNA2, sgRNA3, sgRNA5. Each sgRNA was individually conducted to form RNP. (b-e) The proposed different sgRNA-based RNP complex to recognize target DNA and other interferences, target EBV cfDNA of 0.35 nM, interferences of 2 mM, data represented as mean ± SD, analyzed by two-way ANOVA: *p<0.05, **p<0.01, ***p<0.001, ****p<0.0001, n=3 independent experiments.

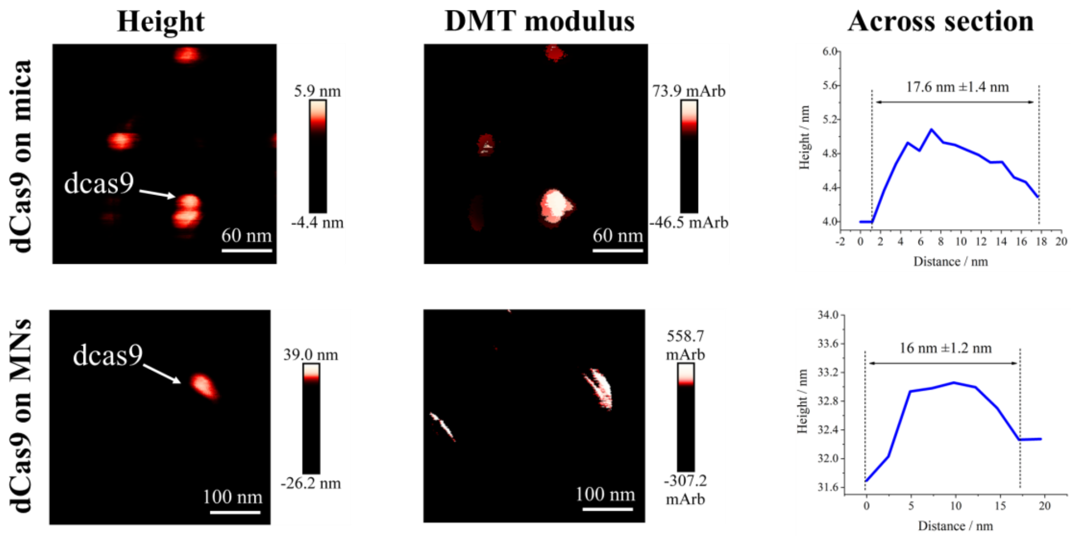

Supplementary Figure 20. Characterization of dCas9 on microneedles by AFM. It indicated that the dCas9 was successfully immobilized on the surface of the microneedles.

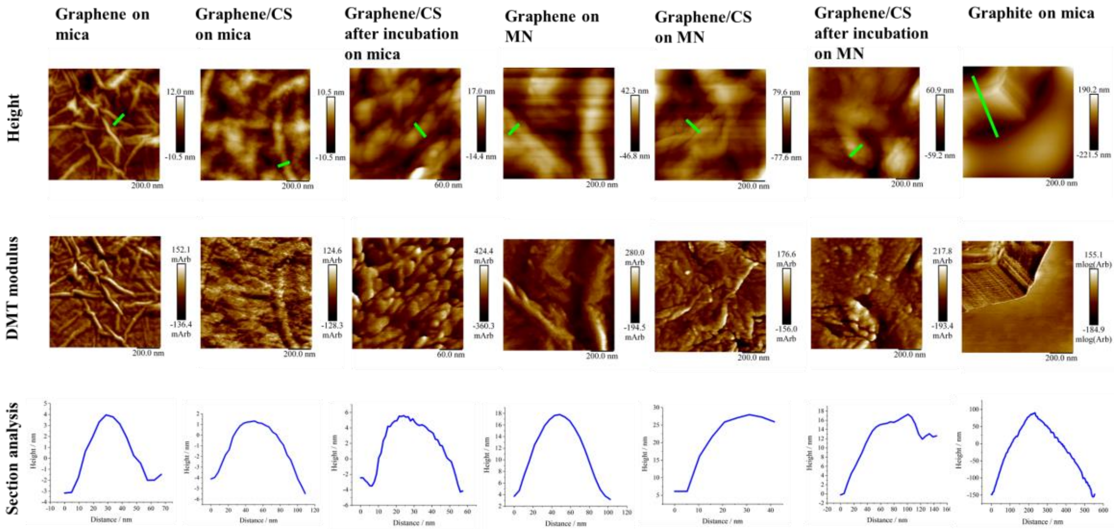

Supplementary Figure 21. AFM observation of graphene nanoflakes on microneedles. The green line represents the regions of section analysis.

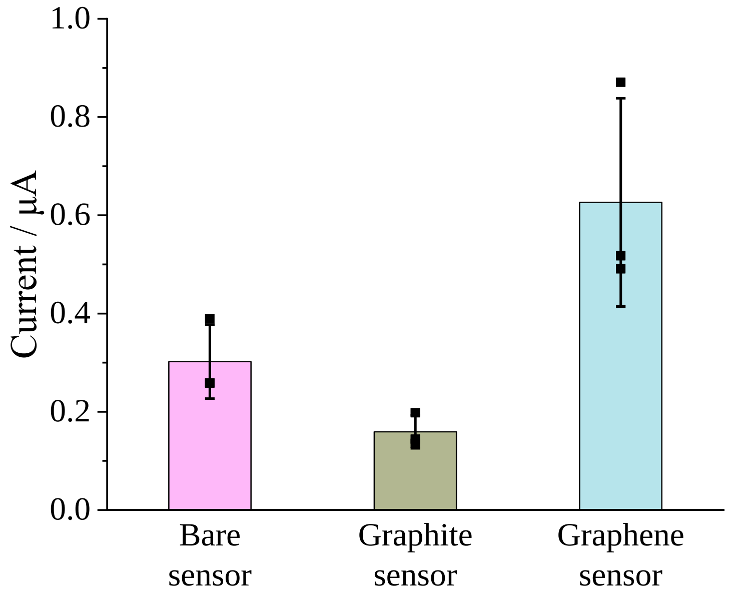

Supplementary Figure 22. The comparison of conductivity for bare sensor, graphite sensor, and graphene sensor. All the sensors were based on a gold electrode (diameter of 2 mm), 0.2 mg/mL graphite dispersion drop-casted on the gold electrode surface and incubated at 60 °C for 30 min to fabricate graphite sensor, 0.2 mg/mL graphene nanoflakes/chitosan dispersion drop casted on the gold electrode surface and incubated at 60 °C for 30 min to fabricate graphene sensor. PBS buffer (0.01 M, pH 7.4); initial potential, 0 V; quiet time, 0 s; sensitivity (A/V), 1×10^{-5} A/V, data presented as mean values ± SD, n=3 independent experiments. The graphene sensor showed a bigger current response compared with graphite sensor.

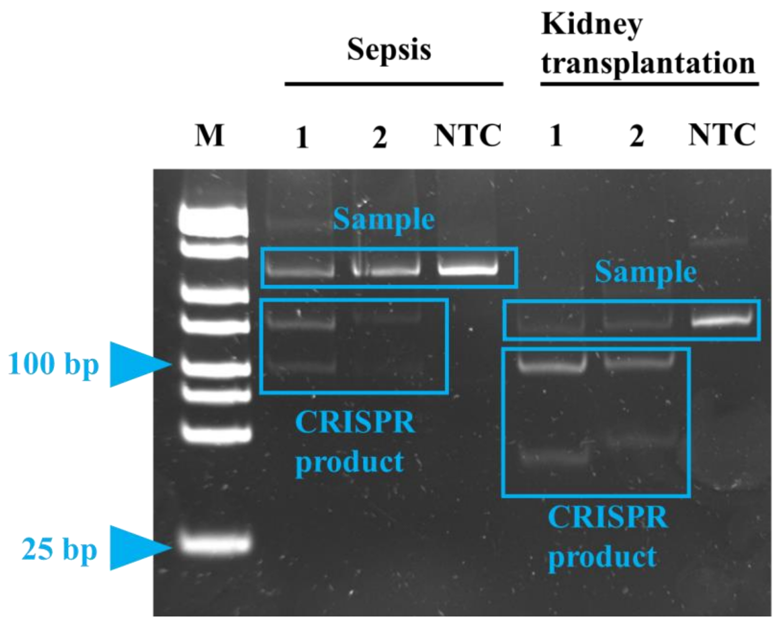

Supplementary Figure 23. Page gel result of optimization for sgRNA targeting to sepsis and kidney transplantation cfDNA. Lane 1, lane 2 referring to sgRNA1 and sgRNA2 respectively, the results obtained from three independent repeated experiments. From the result, sgRNA 1 of sepsis and sgRNA1 of kidney transplantation were optimal, thus they were used for the subsequent experiments.

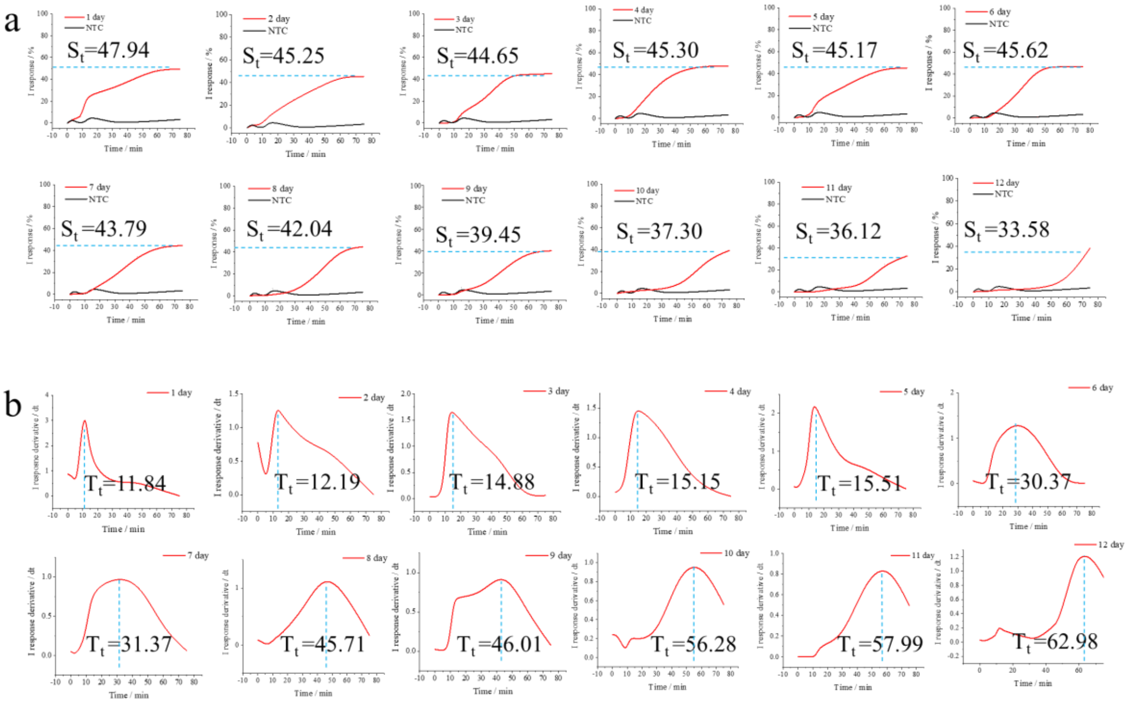

Supplementary Figure 24. Stable sensitivity tests *in vitro*. (a) Real-time signal curves for 12 days, n=3 independent experiments. (b) the relative slope of the plots for 12 days, calculated by simple differentiation, n=3 independent experiments. S_t value was defined as signal platform threshold and T_t value was defined as the time threshold corresponding to the maximum of signal response curve derivatives.

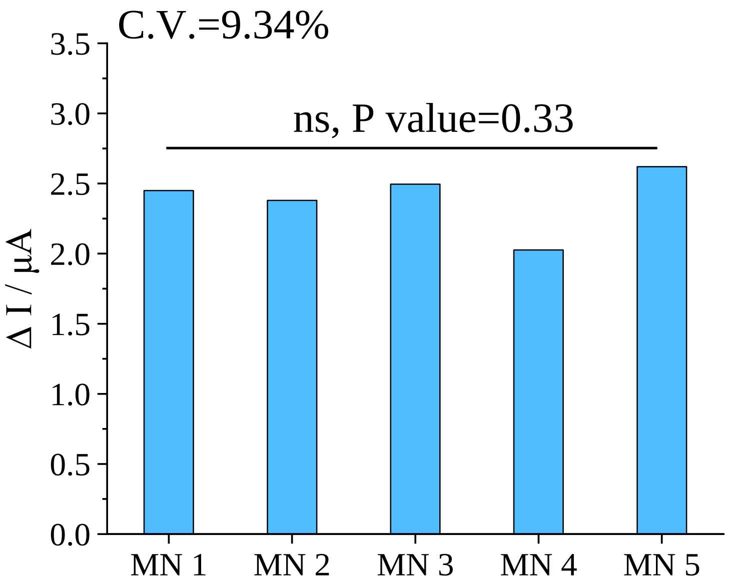

Supplementary Figure 25. Sensor to sensor variation. Current was recorded in the simulated ISF (PBS, 0.01 M, pH 7.4); quiet time, 0 s; sensitivity (A/V), 1×10^{-3} A/V, analyzed by two-way ANOVA, p value=0.33, n=5 independent samples.

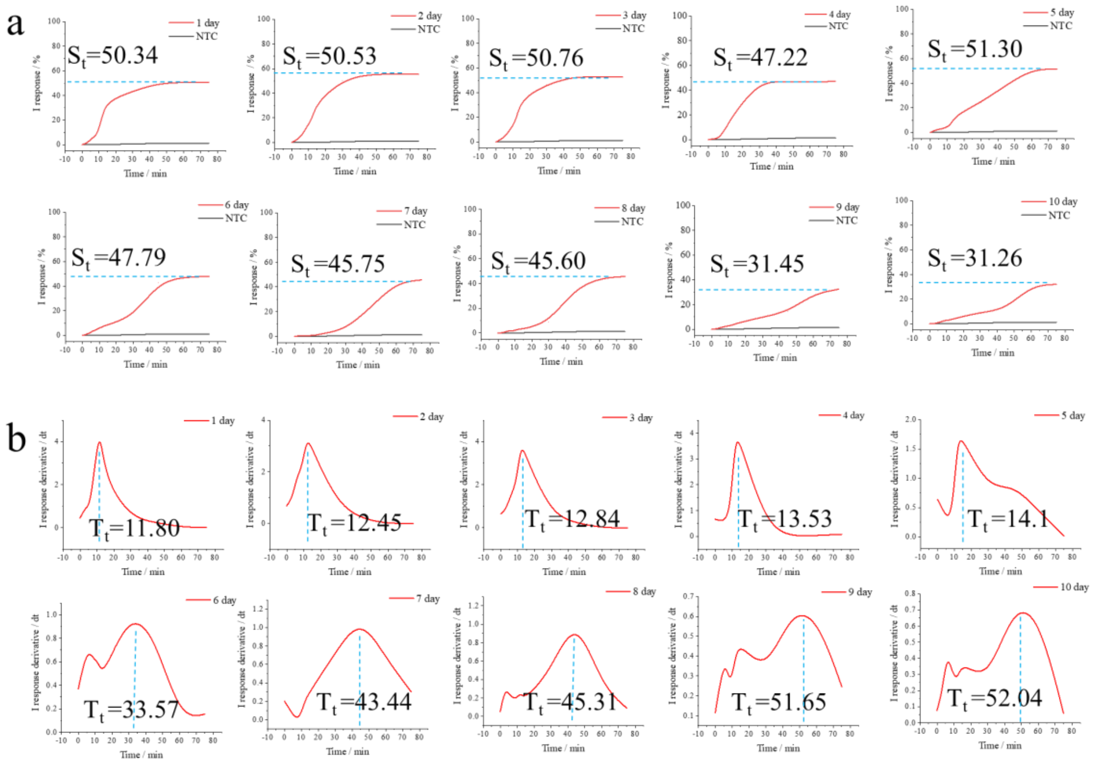

Supplementary Figure 26. Stable sensitivity tests *in vivo*. (a) Real-time signal curves for 10 days, n=3 independent experiments. (b) the relative slope of the plots for 10 days, calculated by simple differentiation, n=3 independent experiments. S_t value was defined as signal platform threshold and T_t defined as the time threshold corresponding to the maximum of signal response curve derivatives.

Supplementary Table 1. CRISPR-Cas MNs compared with other MNs method in the field of chem-biosensing.

Strategy	Analyte	Function	Time	Real-time (Y/N)	Conductive (Y/N)	Ref.
Wearable array	Opioid and organophosphate	Detection	~200 min	Y	Y	[7]
Stainless steel MNs	Caffeine and glucose in ISF	Sampling	~20 min	N	Y	[8]
Gelatin methacryloyl MNs	Glucose and vancomycin in ISF	Sampling	~10 min	N	N	[9]
MNs detection system	Glucose and lactate	Detection	300 min	Y	Y	[10]
Alginate-peptide MNs	MicroRNA	Sampling and detection	2 min	N	N	[11]
PVA MNs	Phytophthora infestans DNA	Sampling	~1 min	N	N	[12]
Photonic crystal-MNs	Inflammatory cytokines in ISF	Sampling and detection	~40 min	N	N	[13]
SERS MNs	pH level in ISF	Detection	NG	N	N	[14]
MNs based on near-infrared quantum dots	Vaccine	Detection	2-5 min	N	N	[15]
rGO-assemble MNs	H_2O_2	Detection	~5 min	Y	Y	[16]
Potentiometric MNs	K^+	Detection	1440 min	Y	Y	[17]

MNs-based biosensor	Lactate	Detection	15 min	Y	Y	[18]
SERS MNs array	Glucose	Detection	15 min	N	N	[19]
Wearable electrochemical MNs	Levodopa	Detection	1 min	Y	Y	[20]
MNs-based Sensor	β-lactam antibiotic	Detection	~330 min	Y	Y	[21]
Porous MNs array	Glucose	Sampling, detection, and drug delivery	~60 min	Y	Y	[22]
CRISPR-Cas MNs	EBV, sepsis, kidney transplantation cfDNA	Sampling and detection	~30 min	Y	Y	This work

Supplementary Table 2. Nucleic acid sequences in the experiments.

	Sequence (5’ - 3’)
RNA 1 of EBV	**CCCGCCUCCAGGCGCCCUCC**GUUUUAGAGCUAGAAAUAGCAAGUUAAAAUAAGGCUAGUCCGUUAUCAACUUGAAAAAGUGGCACCGAGUCGGUGCUUUU
RNA 2 of EBV	**CCCGCCTCCAGGCGCCCUCC**GUUUUAGAGCUAGAAAUAGCAAG
RNA 3 of EBV	**UUCAGAGGAACCAGGGACCU**GUUUUAGAGCUAGAAAUAGCAAG
RNA 4 of EBV	**GAGAGGGGCAGAACCAACCC**GUUUUAGAGCUAGAAAUAGCAAGUUAAAAUAAGGCUAGUCCGUUAUCAACUUGAAAAAGUGGCACCGAGUCGGUGCUUUU
RNA 5 of EBV	**UAAACCCAAAGAAUGUCUGA**GUUUUAGAGCUAGAAAUAGCAAGUUAAAAUAAGGCUAGUCCGUUAUCAACUUGAAAAAGUGGCACCGAGUCGGUGCUUUU
RNA 6 of EBV	**AGACAUUCUUUGGGUUUAAC**GUUUUAGAGCUAGAAAUAGCAAGUUAAAAUAAGGCUAGUCCGUUAUCAACUUGAAAAAGUGGCACCGAGUCGGUGCUUUU
RNA 1 of sepsis	**CCCCUGCUACUGCCAGGACC**GUUUUAGAGCUAGAAAUAGCAAGUUAAAAUAAGGCUAGUCCGUUAUCAACUUGAAAAAGUGGCACCGAGUCGGUGCUUUU
RNA 2 of sepsis	**CUACUGCCAGGACCAGGCCC**GUUUUAGAGCUAGAAAUAGCAAGUUAAAAUAAGGCUAGUCCGUUAUCAACUUGAAAAAGUGGCACCGAGUCGGUGCUUUU
RNA 1 of kidney transplantation	**AUUCAUUCUCAAGCAAAACA**GUUUUAGAGCUAGAAAUAGCAAGUUAAAAUAAGGCUAGUCCGUUAUCAACUUGAAAAAGUGGCACCGAGUCGGUGCUUUU

RNA 2 of kidney transplantation	**AGGACAGCAGUAGAGCAGUC**GUUUUAGAGCUAGAAAUAGCAAGUUAAAAUAAGGCUAGUCCGUUAUCAACUUGAAAAAGUGGCACCGAGUCGGUGCUUUU
EBV plus	TTCCCTCCTTTTGTAAACAATGCATTCATCGACCTTCCAGCCGGGGTTGGTTCTGCCCCTCTCTCTGTCCTTCAGAGGAACCAGGGACCTCGGGCACCCCAGAGCCCCTCGGGCCCGCCTCCAGGCGCCCTCCTGGTCTCCGCTCCCCTCTGAGCCCCGTTAAACCCAAAGAATGTCTGAGGGGAGCCACCCTCGGGGCCCAGGCCCCAGAGTCACACATCCGACACAACAACAGCATTCTCCT
Sepsis	GACCCATCTGGCCGCCTCCCGAGAGGCCATGGGCGCTGTGACTCCTTCATCTTGGCCTAGGAAGCACCAGCCTTCAGCTGCTCACGCCAGATTCTTGCAGACATTGCAACTCCTCTTTTTCTCGGCTCTACCTTCCACAAACATCCCCTGCTACTGCCAGGACCAGGCCCCGGCCCCGATCCCGGCCCGGTCCACCGCAGCCCATCCCCGCACTGGCTCCTTGCTGCCCCCGACCCTCCCAGCAGCCAGAGGGACTTTTCACC
Kidney transplantation	CCCCCTAGTACCCTGACAATGTATTCATTCTCAAGCAAAACATGGTAATTCAGTAACGTTGACTACTTGCCCTGCTGATCTGCCTCCCTGACTGCTCTACTGCTGTCCTGAAAAATGCGAATTTGACTTAATCGCCA
PCR F1 for EBV plus	TGTTGTTGTGTCGGATGTGT
PCR R1 for EBV plus	TTCCAGCCGGGGTTGGTTCT
PCR F2 for EBV plus	GTGTGACTCTGGGGCCTGGGCC

PCR R2<br>for EBV<br>plus	CTCCTTTTGTAAACAATGCATT
PCR F3<br>for EBV<br>plus	GTGTGACTCTGGGGCCTGGGCCCC
PCR R3<br>for EBV<br>plus	CCCTCCTTTTGTAAACAATGCATT
PCR F<br>primer<br>for EBV<br>primer	GGTTCTGCCCCTCTCTCTGTCC
PCR R<br>primer<br>for EBV	AGGGGAGCGGAGACCAGGAG

Supplementary Reference

[1] J. Wang, S. Deng, Z. Liu, Z. Liu, *Natl. Sci. Rev.* **2015**, 2, 22.

[2] P. Bergveld, *Biosens. Bioelectron.* **1991**, 6, 55.

[3] F. A. Ran, P. D. Hsu, J. Wright, V. Agarwala, D. A. Scott, F. Zhang, *Nat. Protoc.* **2013**, 8, 2281.

[4] L. Cong, F. A. Ran, D. Cox, S. Lin, R. Barretto, N. Habib, P. D. Hsu, X. Wu, W. Jiang, L. A. Marraffini, F. Zhang, *Science* **2013**, 339,819.

[5] R. K. Mishra, K. Y. Goud, Z. Li, C. Moonla, M. A. Mohamed, F. Tehrani, H. Teymourian, J. Wang, *J. Am. Chem. Soc.* **2020**, 142, 5991.

[6] L. M. Gan et al., *Nat. Commun.* **2019**, 10, 871.

[7] J. R. Sempionatto et al., *Nat. Biomed. Eng.* **2021**, 5, 737.

[8] P. P. Samant, M. M. Niedzwiecki, N. Raviele, V. Tran, J. M. Lapaix, D. I. Walker, E. I. Felner, D. P. Jones, G. W. Miller, M. R. Prausnitz, *Sci. Transl. Med.* **2020**, 12, eaaw0285.

[9] J. Zhu, X. Zhou, H. J. Kim, M. Qu, X. Jiang, K. Lee, L. Ren, Q. Wu, C. Wang, X. Zhu, P. Tebon, S. Zhang, J. Lee, N. Ashammakhi, S. Ahadian, M. R. Dokmeci, Z. Gu, W. Sun, A. Khademhosseini, *Small* **2020**, 16, 1905910.

[10] H. Teymourian, C. Moonla, F. Tehrani, E. Vargas, R. Aghavali, A. Barfidokht, T. Tangkuaram, P. P. Mercier, E. Dassau, J. Wang, *Anal. Chem.* **2020**, 92, 2291.

[11] D. A. Sulaiman, J. Y. H. Chang, N. R. Bennett, H. Topouzi, C. A. Higgins, D. J. Irvine, S. Ladame, *ACS nano* **2019**, 13, 9620.

[12] R. Paul, A. C. Saville, J. C. Hansel, Y. Ye, C. Ball, A. Williams, X. Chang, G. Chen, Z. Gu, J. B. Ristaino, Q. Wei, *ACS nano* **2019**, 13, 6540.

[13] X. Zhang, G. Chen, F. Bian, L. Cai, Y. Zhao, *Adv. Mater.* **2019**, 31, 1902825.

[14] J. E. Park, N. Y. Tanyeri, E. V. Ende, A. I. Henry, B. E. P. White, M. Mrksich, R. P. V. Duyne, *Nano Lett.* **2019**, 19, 6862.

[15] K. J. McHugn, L. Jing, S. Y. Severt, M. Cruz, M. Sarmadi, H. S. N. Jayawardena, C. F. Perkinson, F. Larusson, S. Rose, S. Tomasic, T. Graf, S. Y. Tzeng, J. L. Sugarman, D. Vlasic, M. Peters, N. Peterson, L. Wood, W. Tang, J. Yeom, J. Collins, P. A. Welkhoff, A. Karchin, M. Tse, M. Gao, M. G. Bawendi, R. Langer, A. Jaklenec, *Sci. Transl. Med.* **2019**, 11, eaay7162.

[16] Q. Jin, H. J. Chen, X. Li, X. Huang, Q. Wu, G. He, T. Hang, C. Yang, Z. Jiang, E. Li, A. Zhang, Z. Lin, F. Liu, X. Xie, *Small* **2019**, 15, 1804298.

[17] M. Parrilla, M. Cuartero, S. P. Sánchez, M. Rajabi, N. Roxhed, F. Niklaus, G. A. Crespo, *Anal. Chem.* **2019**, 91, 1578.

[18] P. Bollella, S. Sharma, A. E. G. Cass, R. Antiochia, *Biosens. Bioelectron.* **2019**, 123,152.

[19] J. Ju, C. M. Hsieh, Y. Tian, J. Kang, R. Chia, H. Chang, Y. Bai, C. Xu, X. Wang, Q. Liu, *ACS Sens.* **2020**, 5, 1777.

[20] K. Y. Goud, C. Moonla, R. K. Mishra, C. Yu, R. Narayan, I. Litvan, J. Wang, *ACS Sens.* **2019**, 4, 2196.

[21] S. A. N. Gowers, D. M. E. Freeman, U. M. Rawson, M. L. Rogers, R. C. Wilson, A. H. Holmes, A. E. Cass, D. O'Hare, *ACS Sens.* **2019**, 4, 1072.

[22] S. Kusama, K. Sato, Y. Matsui, N. Kimura, H. Abe, S. Yoshida, M. Nishizawa, *Nat. Commun.* **2021**, 12, 658.
