# Supplementary material for: Programmable CRISPR-Cas9 microneedle patch for long-term capture and real-time monitoring of universal cell-free DNA
Source: Nat Commun. 2022 Jul 9;13:3999. doi: 10.1038/s41467-022-31740-3 (PMC9271037; doi:10.1038/s41467-022-31740-3)
Supplement: Supplementary file 3 — Description of additional Supplementary File [file 41467_2022_31740_MOESM3_ESM.pdf]

Descriptions of additional Supplementary Data files

Supplementary Movie 1 is ‘The printed wearable patch in the static’.

Supplementary Movie 2 is ‘The wearable patch for human physical activity’.

Supplementary Movie 3 is ‘The application of the wearable CRISPR microneedles in real-world’.
