# Supplementary material for: Programmable CRISPR-Cas9 microneedle patch for long-term capture and real-time monitoring of universal cell-free DNA
Source: Nat Commun. 2022 Jul 9;13:3999. doi: 10.1038/s41467-022-31740-3 (PMC9271037; doi:10.1038/s41467-022-31740-3)
Supplement: Supplementary file 7 — Reporting Summary [file 41467_2022_31740_MOESM7_ESM.pdf]

nature research

Corresponding author(s): Xueen Fang and Jilie Kong

Last updated by author(s): Jun 11, 2022

Reporting Summary

Nature Research wishes to improve the reproducibility of the work that we publish. This form provides structure for consistency and transparency in reporting. For further information on Nature Research policies, see our Editorial Policies and the Editorial Policy Checklist.

Statistics

For all statistical analyses, confirm that the following items are present in the figure legend, table legend, main text, or Methods section.

n/a	Confirmed	
☐	☒	The exact sample size (n) for each experimental group/condition, given as a discrete number and unit of measurement
☐	☒	A statement on whether measurements were taken from distinct samples or whether the same sample was measured repeatedly
☐	☒	The statistical test(s) used AND whether they are one- or two-sided<br>*Only common tests should be described solely by name; describe more complex techniques in the Methods section.*
☒	☐	A description of all covariates tested
☒	☐	A description of any assumptions or corrections, such as tests of normality and adjustment for multiple comparisons
☐	☒	A full description of the statistical parameters including central tendency (e.g. means) or other basic estimates (e.g. regression coefficient) AND variation (e.g. standard deviation) or associated estimates of uncertainty (e.g. confidence intervals)
☐	☒	For null hypothesis testing, the test statistic (e.g. F, t, r) with confidence intervals, effect sizes, degrees of freedom and P value noted<br>*Give P values as exact values whenever suitable.*
☒	☐	For Bayesian analysis, information on the choice of priors and Markov chain Monte Carlo settings
☒	☐	For hierarchical and complex designs, identification of the appropriate level for tests and full reporting of outcomes
☒	☐	Estimates of effect sizes (e.g. Cohen's d, Pearson's r), indicating how they were calculated

Our web collection on statistics for biologists contains articles on many of the points above.

Software and code

Policy information about availability of computer code

Data collection	The mechanical testing of microneedle patch was recorded via a stylus profiler (AlphaStep D-600, KLA-Tencor Corp.) and electronic universal testing machine (Instron 5966, USA). The page gel electrophoresis of CRISPR-Cas9 was imaged by EPS 300 and 4100 digital gel image system (Tannon Corp.). All the mechanical testing of the wearable PDMS patch was performed on Instron 5966 electronic universal testing machine (Instron, USA). PCR was performed on a fluorescent quantitative PCR detection system (LineGene 9640, Hangzhou Bioer Technology Co., Ltd., Hangzhou, China). All the electrochemical characterization, acquisition, and testing was performed with a electrochemical workstation CHI 1030 and autolab (Nova 1.7). Contact angle was conducted on JC2000D (Powereach Co.). SEM was conducted on Zeiss Gemini SEM500 FESEM and VEGA 3 XMU (TESCAN Co., Czech). Animal bioimaging was performed with in Vivo Xtreme (Bruker, USA). Finite element analysis of the wearable patch was collected by COMSOL Multiphysics 5.3.
Data analysis	All the electrochemical data was analyzed by Origin 2018 software and Zview software. Microsoft Excel 2016 was used to analyze mechanical testing data and contact angle.The next-generation sequencing was analyzed by Chromas version 2.3 software. And all the primers was analyzed by Primer premiere 5.0 and GeneRunner 6.5.51 software. The images of PAGE gel results were analyzed by Image J 1.51K. All the animal bioimaging results were analyzed by Bruke MI SE.

For manuscripts utilizing custom algorithms or software that are central to the research but not yet described in published literature, software must be made available to editors and reviewers. We strongly encourage code deposition in a community repository (e.g. GitHub). See the Nature Research guidelines for submitting code & software for further information.

Data

Policy information about availability of data

All manuscripts must include a data availability statement. This statement should provide the following information, where applicable:

- Accession codes, unique identifiers, or web links for publicly available datasets
- A list of figures that have associated raw data
- A description of any restrictions on data availability

The GenBank data in this study have been deposited in the NCBI database under accession code NO. M12294.2 [https://www.ncbi.nlm.nih.gov/nuccore/11497619], NO. NC001437.1 [https://www.ncbi.nlm.nih.gov/nuccore/NC_001437], NO. AF326573.1 [https://www.ncbi.nlm.nih.gov/nuccore/AF326573.1], NO. A10072.1 [https://www.ncbi.nlm.nih.gov/nuccore/A10072.1], NO. NC_000024.10 [https://www.ncbi.nlm.nih.gov/nuccore/NC_000024.10] for WENV, JPEV, DENV, EBV, kidney transplantation, respectively. All the data generated and analyzed in the study are included in the paper and supplementary information. The source data underlying Fig. 2a, 2c-2l, 3b-3f, 3l-3o, 4c-4e, 5b-5j, 6c-e, 6g and Supplementary Fig. 1c, 2b-2e, 4, 5, 6, 7a, 7b, 8d, 11, 12, 14, 15, 16c, 17, 19, 20, 21, 24, 25, and 26 are provided as a Source Data file. Source data are provided with this paper.

Field-specific reporting

Please select the one below that is the best fit for your research. If you are not sure, read the appropriate sections before making your selection.

☒ Life sciences ☐ Behavioural & social sciences ☐ Ecological, evolutionary & environmental sciences

For a reference copy of the document with all sections, see nature.com/documents/nr-reporting-summary-flat.pdf

Life sciences study design

All studies must disclose on these points even when the disclosure is negative.

Sample size	For animal experiment, the 4-week-old female Balb/c nude mice (n=3 for each group) and 4-week-old female KM mice (n=3 for each group) were purchased from Beijing Vital River Laboratory Animal Technology Co., Ltd. (Beijing, China), whose sample sizes were sufficient for two-way ANOVA analysis in the study. For human trials, the consenting subjects were healthy (19-37 years old, total number of 10), whose sample sizes were sufficient for significant difference analysis and two-way ANOVA analysis.
Data exclusions	No data were excluded
Replication	Data acquisition was conducted on different groups (n=3 for each group) for one experimental performance to verify the feasibility of the device. And three experimental performances were conducted in different period of time. The time interval of each experimental performance was 1 month.
Randomization	The BALB/c nude mice and KM mice with different activity levels were randomly allocated into the groups. For human trials, participants with different self-reported activity level were randomly recruited.
Blinding	No blinding measures were taken deliberately for Balb/c nude mice and KM mice. Because those Balb/c nude mouse with NPC tumour were used as positive models and healthy Balb/c nude mouse were used as negative models, which provided practical models for the following experiments. And for KM mice, because we chose healthy KM mice to offer a epidermis microenvironment for the verification of the device in vivo. All the animal experiment data was processed together by multiple research group members. For human trials, we randomly recruited participants with different self-reported activity level, and we didn't know their genetic disease.

Reporting for specific materials, systems and methods

We require information from authors about some types of materials, experimental systems and methods used in many studies. Here, indicate whether each material, system or method listed is relevant to your study. If you are not sure if a list item applies to your research, read the appropriate section before selecting a response.

Materials & experimental systems

n/a	Involved in the study	
☒	☐	Antibodies
☐	☒	Eukaryotic cell lines
☒	☐	Palaeontology and archaeology
☐	☒	Animals and other organisms
☐	☒	Human research participants
☒	☐	Clinical data
☒	☐	Dual use research of concern

Methods

n/a	Involved in the study	
☒	☐	ChIP-seq
☒	☐	Flow cytometry
☒	☐	MRI-based neuroimaging

Eukaryotic cell lines

Policy information about cell lines

Cell line source(s)	CNE cells (Catalog NO. BNCC341794) and HUVECS cells (Catalog NO. BNCC337616) were purchased from Beina Chuanglian Biology Research Institute (Beijing, China). HACAT cells (Catalog NO. CL-0090) and HFF cell (Catalog NO. ZQ0450) were purchased from Procell Life Science & Technology Co., Ltd. (Wuhan, China) and Shanghai Zhong Qiao Xin Zhou Biotechnology Co., Ltd. (Shanghai, China), respectively. HeLa-GFP cells (HeLa Kyoto EGFP-H2B, Catalog NO. 300673) were purchased from Cell Line Service (CLS, Germany). And all the cell lines were authenticated by cells supplier incorporations.
Authentication	All the cell lines were not authenticated by the authors. And all the cell lines were authenticated by cells supplier incorporations.
Mycoplasma contamination	Mycoplasma tests were conducted and the results were negative.
Commonly misidentified lines (See ICLAC register)	No commonly misidentified cell lines were used

Animals and other organisms

Policy information about studies involving animals; ARRIVE guidelines recommended for reporting animal research

Laboratory animals	4-week-old female Balb/c nude mice and 4-week-old female KM mice were in the same housing condition, which was in the cycle of 6-hour dark/18-hour light (23 °C±2°C, 30%-40% of ambient humidity).
Wild animals	The study did not involve wild animals
Field-collected samples	The study did not involve the collected samples from field.
Ethics oversight	Animals were cared for and maintained under the Guidelines of Laboratory Animals of Fudan University and approved by the Animal Ethics Committee of Fudan University, China.

Note that full information on the approval of the study protocol must also be provided in the manuscript.

Human research participants

Policy information about studies involving human research participants

Population characteristics	Healthy Subject (female and male, 19-37 years).
Recruitment	Samples were randomly recruited from research group members in the age of 19-37 years old (5 female participants and 5 male participants). They are with different self-reported activity level, and we didn't know their genetic disease. Maybe some of them have genetic skin diseases or others, which might have impact on the results.
Ethics oversight	The human research was approved by Ethics Committee of Fudan University and complied with all relevant ethical regulations (IRB NO. FE20037). The informed consents were given by human participants, and they were compensated.

Note that full information on the approval of the study protocol must also be provided in the manuscript.
